# T cell receptor–centric perspective to multimodal single-cell data analysis

**DOI:** 10.1126/sciadv.adr3196

**Published:** 2024-11-29

**Authors:** Kerry A. Mullan, My Ha, Sebastiaan Valkiers, Nicky de Vrij, Benson Ogunjimi, Kris Laukens, Pieter Meysman

**Affiliations:** ^1^ Adrem Data Lab, Department of Computer Science, University of Antwerp, Antwerp, Belgium.; ^2^ Antwerp Unit for Data Analysis and Computation in Immunology and Sequencing (AUDACIS), Antwerp, Belgium.; ^3^ Biomedical Informatics Research Network Antwerp (Biomina), University of Antwerp, Antwerp, Belgium.; ^4^ Antwerp Center for Translational Immunology and Virology (ACTIV), Vaccine and Infectious Disease Institute, University of Antwerp, Antwerp, Belgium.; ^5^ Centre for Health Economics Research and Modelling Infectious Diseases (CHERMID), Vaccine and Infectious Disease Institute, University of Antwerp, Antwerp, Belgium.; ^6^ Clinical Immunology Unit, Department of Clinical Sciences, Institute of Tropical Medicine, Antwerp, Belgium.; ^7^ Department of Paediatrics, Antwerp University Hospital, Antwerp, Belgium.

## Abstract

The T cell receptor (TCR), despite its importance, is underutilized in single-cell analysis, with gene expression features solely driving current strategies. Here, we argue for a TCR-first approach, more suited toward T cell repertoires. To this end, we curated a large T cell atlas from 12 prominent human studies, containing in total 500,000 T cells spanning multiple diseases, including melanoma, head and neck cancer, blood cancer, and lung transplantation. Here, we identified severe limitations in cell-type annotation using unsupervised approaches and propose a more robust standard using a semi-supervised method or the TCR arrangement. We showcase the utility of a TCR-first approach through application of the STEGO.R tool for the identification of treatment-related dynamics and previously unknown public T cell clusters with potential antigen-specific properties. Thus, the paradigm shift to a TCR-first can highlight overlooked key T cell features that have the potential for improvements in immunotherapy and diagnostics.

## Teaser

T cell receptor–centric perspective improves single-cell analysis.

## INTRODUCTION

T cells are specialized cells of the adaptive immune system positioned to recognize various infected or otherwise aberrant cells. This recognition is driven by the T cell receptor (TCR) expressed on the cellular surface. Their importance for the immune response, and various underlying disease states, have driven recent innovations in the domain of single-cell sequencing. We can now capture a multimodal view of a single T cell that includes the transcription gene expression (GEx) and the hypervariable TCR (*1*). This detailed perspective allows distinction of the T cell population into its phenotypes with the cognate TCRs. The broadest phenotypic distinction is between CD4^+^ T cells that recognize the class II major histocompatibility complex (MHC) loaded with extracellular peptide sources and CD8^+^ T cells that recognize the class I MHC loaded with intracellular peptides. CD4^+^ T cells exhibit diverse functional phenotypes, categorized into T helper (T_H_) subtypes such as T_H_1, T_H_2, T_H_9, T_H_17, T follicular T helper (T_FH_) cells, and regulatory T cells (T_regs_). T cells also display a variety of memory states and can transition from activation to senescence or exhaustion. In addition to their expression markers, T cells are divided into two principal lineages based on the TCR, namely, alpha-beta (αβ) or gamma-delta (γδ). Each of these four TCR chains is made up of a variable (V), diversity (D), and junction (J) genes, with α/γ having VJ and β/δ VDJ arrangements. During development a T cell will acquire a quasi-random recombination of V(D)J genes, which results in a hypervariable sequence in the complementarity determining region 3 (CDR3), the primary driver of variable antigen specificity. Recent methodological advances have enhanced the analysis of bulk TCR repertoire data, with tools based on identifying antigen-specific TCR groups [e.g., ClusTCR (*2*), tcrdist3 (*3*), GIANA (*4*), and GLIPH (*5*)] and epitope annotation models [TCRex (*6*), ImmuneWatch DETECT, NetTCR (*7*), and mixTCRpred (*8*)]; however, thus far, these approaches have had limited adoption for single-cell data (*5*–*8*).

The current state of the single-cell field uses standardized pipelines and focuses on interrogating unsupervised-based annotations. However, this “GEx-centric” approach may not be the most ideal methodology for every field. With T cells, the critical interrogation challenge involves the analysis of single-cell data with not one but two key data levels. Conventionally, data analysis pipelines have been developed for bulk TCR sequencing (TCR-seq) (*9*) and GEx experiments independently. Because of the historical development of GEx and TCR-specific analysis tools, there is a disconnect between these two data levels as they are analyzed separately rather than concurrently. Some efforts have been made to leverage both modalities, including CoNGA (*10*) and mvTCR (*11*). Those approaches that do integrate both levels build on the assumption that they are alternative representations of the same information, i.e., one TCR translates to one function (*10*), and does not consider the dynamic states of T cell subpopulation (*12*). Most analysis pipelines simply extend on the existing methods for GEx-centric analysis [e.g., Seurat (*13*), scanpy (*14*), and DALI (*15*)], with the TCR often seen as a secondary aspect to annotate the GEx clusters by using is as an index marker for clonal expansion (*16*).

The GEx-centric approach therefore relies on the accuracy of T cell phenotyping across the unsupervised clustering. However, T cell subpopulation differences are driven by subtle dynamic variations (*17*). This information may not be captured in low-dimensional projections of the GEx matrix, which is used to inform clustering of cell populations (*16*). However, there has been little interrogation using the captured TCR to confirm the accuracy of the unsupervised sell subtype annotations.

Overall, the current GEx-centric analysis is now ignoring a wealth of data within the captured TCR-seq layer. Thus, we aim to create a “field-centric” methodology we defined as “TCR-first” to overcome the generic GEx limitations. Here, we compiled a T cell atlas of 12 publicly available datasets containing ~500,000 cells with both TCR and GEx and aimed to determine the utility of a TCR-first analysis. This required the development of a software tool coined “Single-cell T cell receptor and Expression Grouped Ontologies” (STEGO.R) to resolve many of the described analysis issues. Switching to a TCR-first approach, allows identification of (i) dynamic T cell states within a single clone across treatments, (ii) epitope/disease-specific signatures, and (iii) functional similarity between T cells from different lineages. These findings resulted in a unique single T cell atlas, covering the general T cell phenotypes (T_H_1, T_H_2, CD8^+^ effector, etc.), as well as state-specific models e.g., cell cycling, immune checkpoint, cytotoxicity, and T_H_1 cytokine expression along with a TCR-seq–based annotation. Overall, we demonstrate that it was beneficial to have field-centric analysis consideration with this TCR-first approach.

## RESULTS

### Current depth of TCR-seq analysis with GEx-centric approach

To showcase the current limitations of the GEx-centric approach in T cell research, we briefly summarize how the TCR information was described in each of the 12 papers included in the atlas (table S1). Briefly, 11 of 12 were analyzed in R using Seurat with 10 using scRepertoire that categorized the data into count categories of clonal expansion; none of the papers considered clonal expansion normalized as frequency. GSE185659 (*18*), a transplantation focused dataset pre- and post-glucocorticoid treatment, was the only one to compare the top four clones compared to the remaining cells. Two studies, GSE180268 (*19*) and GSE145370 (*20*), used the trajectory modeling of Monocle 3 (*21*). Only GSE180268 head and neck cancer T cell describing them transitioning from stem-like to intermediate profile and finally terminally differentiated state. Overall, the TCR-seq was underused across the 12 studies as the typical GEx-centric approach does not allow for in-depth interrogation of the TCR.

### Processing the data through the STEGO.R tool

**Fig. 1.**
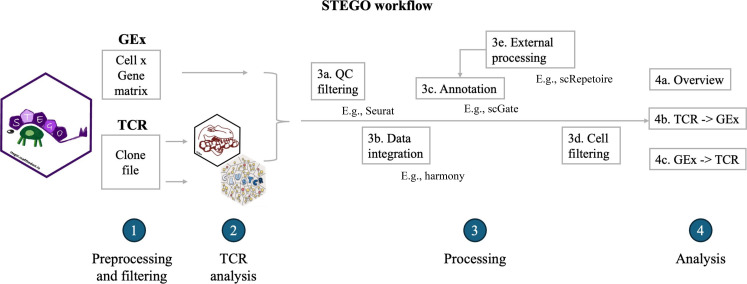
Flow chart representing the quality control process in STEGO.R. The process proceeds from left to right, starting with (**1**) reading and converting the GEx and TCR-seq input files, followed by (**2**) additional TCR processing to add epitope annotation and clustering. The data then undergo (**3**) strict filtering by removing low-quality cells, integrating the data sources, and adding cellular annotation. Last, the data are ready to be analyzed in detail starting from a given TCR clonotype or annotated phenotype (**4**).

The GEx and TCR-seq data from the 12 papers were processed using STEGO (Fig. 1). Briefly, the matrix data were processed and filtered to retain only those cells with both data modalities (step 1). TCR sequences from each clonotype were clustered and epitope annotation was added (step 2). The datasets were combined into a single atlas with batch correction using Harmony before applying the semi-supervised T cell phenotype annotation (step 3). This resulted in a T cell atlas containing 493,784 cells based on 149 unique samples from 90 different individuals.

### T cell atlas identified background data was needed to improve FindMarker enrichment

The datasets were combined into a single atlas, where Harmony was used for the batch correction. We also limited the data to the TCR-GEx as these were high-confidence T cells. In addition, we hypothesized that combining the 12 datasets would improve overall annotations, especially with identifying rarer T cell phenotype and/or study-specific signatures. Originally, all the datasets were analyzed separately; however, we identified a key issue regarding the process when using the Seurat “FindMarker,” which was found to be dependent on similarity to the background.

**Fig. 2.**
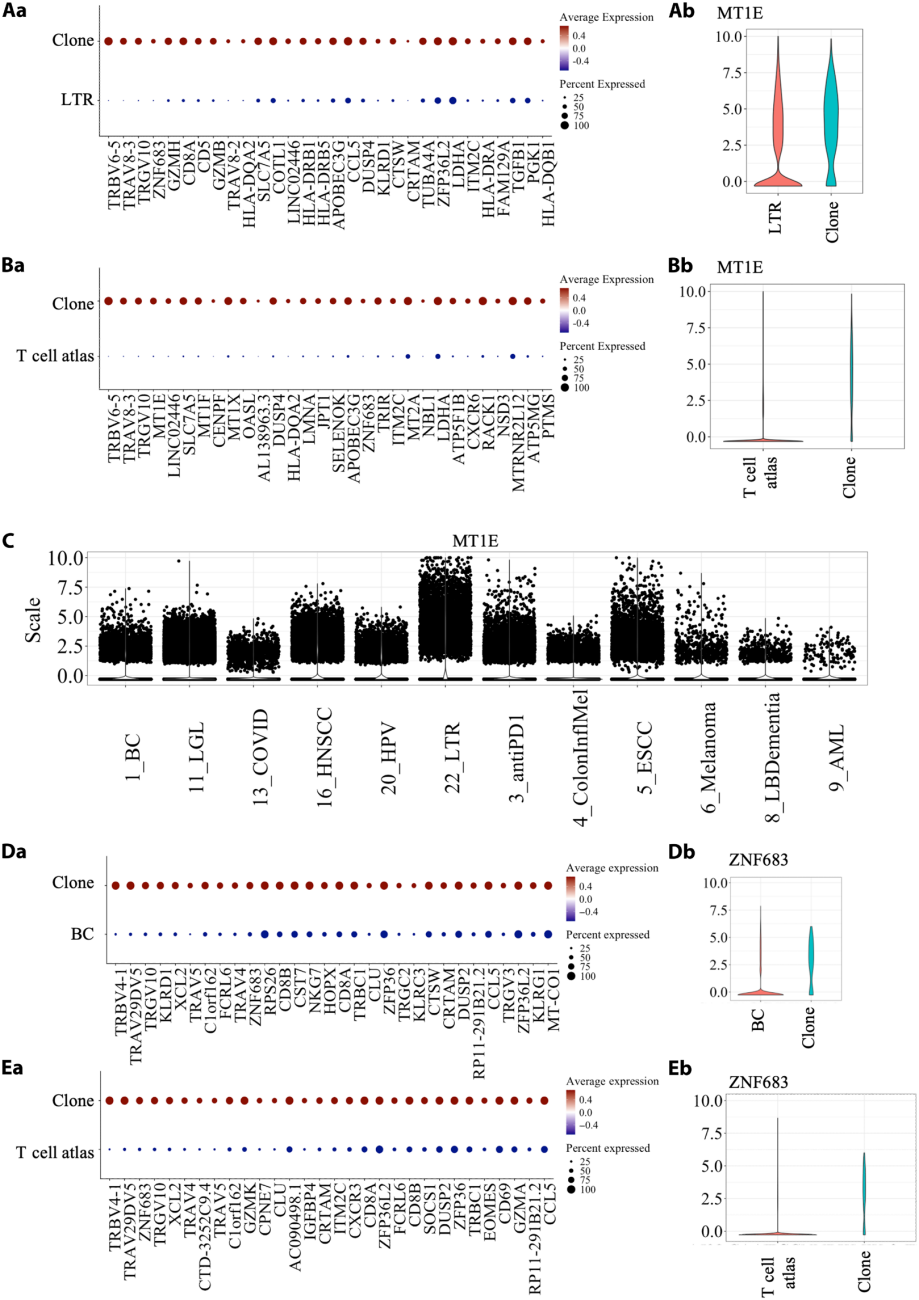
Genes enriched in the lung transplantation recipient (LTR) dataset. (**A** and **B**) Dot plot representing the normalized average expression and the approximant number of cells being expressed comparing the clone of interest TRAV8-3.TRAJ17 CAVGASKAAGNKLTF and TRBV6-5.TRBJ1-5 CASRRTGRNQPQHF to (A) the remaining LTR data and the (B) remaining T cell atlas. (a) Dot plots of the significantly enriched genes that showcase the percentage expressed and the relative expression. (b) Violin plot of the [(A) and (B)] MT1E or [(**D**) and (**E**)] ZNF683 transcript. (**C**) Expression of MT1E across the 12 studies that showcase the range of expression. BC, breast cancer.

To illustrate the annotation issue, we selected to use the lung transplantation recipient (LTR) dataset [GSE185659 (*18*)]. This was one of the few noncancer datasets and therefore should have a distinct profile from the cancer-based disease settings. We aimed to identify if having a larger background dataset would drive out disease specific signature in LTR. The top expanded clone TRAV8-3.TRAJ17 CAVGASKAAGNKLTF and TRBV6-5.TRBJ1-5 CASRRTGRNQPQHF was selected as it was present in acute cellular rejection (ACR) (*n* = 192) and post-glucocorticoid therapy (*n* = 2) for a pairwise analysis. This clone appeared to respond to therapy as it was depleted after glucocorticoid treatment. We compared this clone to the remainder of the LTR dataset (Fig. 2A) and the T cell atlas (Fig. 2B). The clone profile versus the LTR dataset showed a more generic cytotoxic Granzyme B (GZMB) and activation profile [several human leukocyte antigen–DR isotype (HLA-DR) transcripts]. When this clone enrichment was done with the T cell atlas identified several district transcripts including metallothionein 1E (MT1E), which appears to have a role as a regulator of T cell function in certain T cell subsets (*22*). MT1E was not detected to be different in the original analysis as it was widely expressed LTR dataset (Fig. 2Ab). To showcase that this was not because of a sequencing bias, we visualized that MT1E was expressed to varying degrees across all 12 studies (Fig. 2C). Similar issues were observed with the breast cancer (BC) dataset, where we analyzed the top 30 associated genes for the most expanded clones in patient 10 as compared against the BC dataset (Fig. 2D) and against the T cell atlas (Fig. 2E). The ZNF683 (zinc finger protein 683) was a more prominent marker for this clone when comparing against the atlas. We also noted a decrease in the prominence of natural killer (NK) cell lectin-like receptor (KLR) that is associated with NK-like cells when comparing to the atlas. This interrogation highlights the issue of similarity to background, which may mask important disease-specific signals. Therefore, we have made our T cell atlas available to reduce background issues that can occur in small and/or sorted datasets.

### TCR-first approach identified dynamic T cell signatures previously hidden from the GEx-centric approach

To understand the impact of therapy on T cells, there were two datasets within the T cell atlas that included this study format: The T cell lymphoma (GSE168859) (*23*) and the LTR (GSE185659) (*18*).

We first focused on the LTR that had three LTR (P1, P3, and P8) patients that experienced ACR and were treated with glucocorticoids. We aimed to understand how the expanded clones were differentially affected by the treatment. To do this, we evaluated which TCRs were present before and after treatment in LTR and then filtered for those comprising at least 1% of the repertoire in either sample and showing at least a twofold difference after treatment (table S2).

**Fig. 3.**
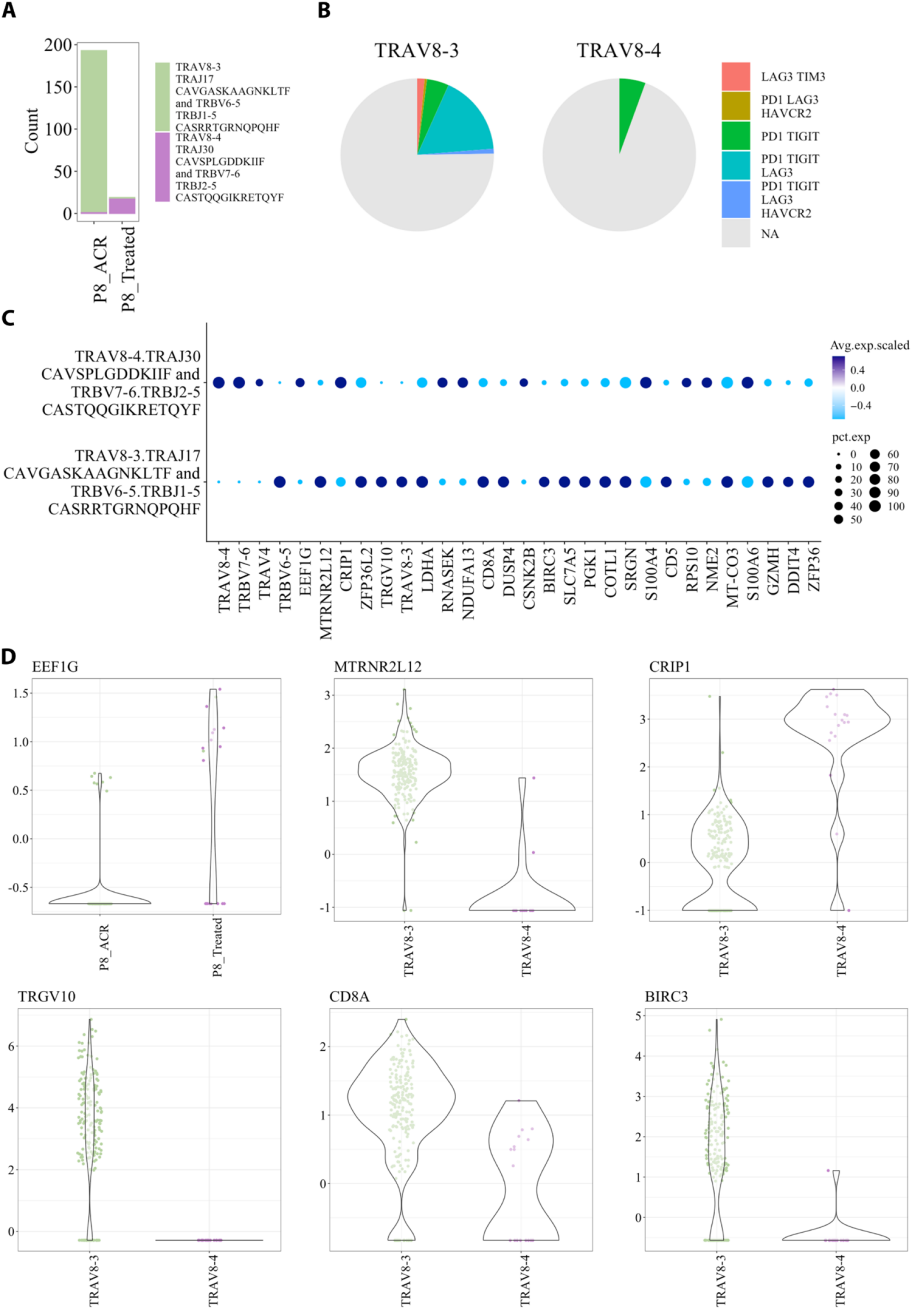
Changes in transcriptional expression during ACR and post-glucocorticoid treatment. (**A**) Total clones for each of the one ACR specific clone (TRAV8-3) and after glucocorticoid treatment (TRAV8-4). (**B**) Expression of two or more immune checkpoints TIGIT, PD1, HAVCR2, and TIM3. (**C**) Differential expression plot comparing the transcriptional signature of ACR to treated. (**D**) Extracted the scaled expression of the transcripts EEF1G, MTRNR2L12, CR1P1, TRGV10, CD8A, and BIRC3. The two clones were from P8.

We examined the top clones that differed the most between ACR and treatment that were present in P8. These were TRAV8-4.TRAJ30_CAVSPLGDDKIIF and TRBV7-6.TRBJ2-5_CASTQQGIKRETQYF with ~65× more in treatment (*n* = 17) versus ACR (*n* = 1) and TRAV8-3.TRAJ17_CAVGASKAAGNKLTF and TRBV6-5.TRBJ1-5_CASRRTGRNQPQHF with ~25× more clones in ACR (*n* = 192 clones) than treated (*n* = 2). As there were less than three counts in one of the time points, there were insufficient cells for statistical analysis for these clones. Instead, we compared the transcriptional signature of these two clones against each other (Fig. 3A). The P8 ACR-specific TCR (TRAV8-3) had more cells with immune checkpoint markers expressed than the treatment-specific clone (TRAV8-4) (Fig. 3B). We next performed marker enrichment analysis comparing these two clones (Fig. 3C) and extracted several of the transcripts that were scaled (most variable across the Atlas). EEF1G and CRIP1 were up-regulated in treatment, and MTRNR2L12, TRGV10, and BIRC3 were almost exclusively expressed in the ACR. CD8A was expressed in both but with higher overall expression in the ACR clone (TRAV8-3) (Fig. 3D).

The other dataset in the T cell atlas that was designed with before and after treatment samples was a large granular lymphocyte (LGL) leukemia dataset. We extracted the likely malignant T cell of the partial responders to the anti-CD52 (alemtuzumab) UPN4 and identified a possible reason for lack of response to treatment (section S2 R1). Both studies identified MTRNR2L12, which was specific to ACR and identified in the post treatment in the UPN4.

### Using TCR-seq for classification and identification of T cell population of interest

Now, even when both GEx and TCR-seq data are available, only the GEx is used for annotation purposes. For instance, mucosal‐associated invariant T (MAIT) cells traditionally have been identified strictly based on GEx features using the expression of TRAV1-2, KLRB1, and/or SLC4A10 (*24*). However, this definition alone may be insufficient to identify the MAIT cells. We replicated this process in the T cell atlas and identified one cluster that had these MAIT-associated markers. We next identified that this MAIT cluster was mostly specific to the Lewy body dementia dataset. However, when leveraging the TCR-seq classification, which uses the variable TRAV1-2 and junction TRAJ33/20/12 genes, we identified possible MAIT cells in every dataset (section S2 R3).

**Fig. 4.**
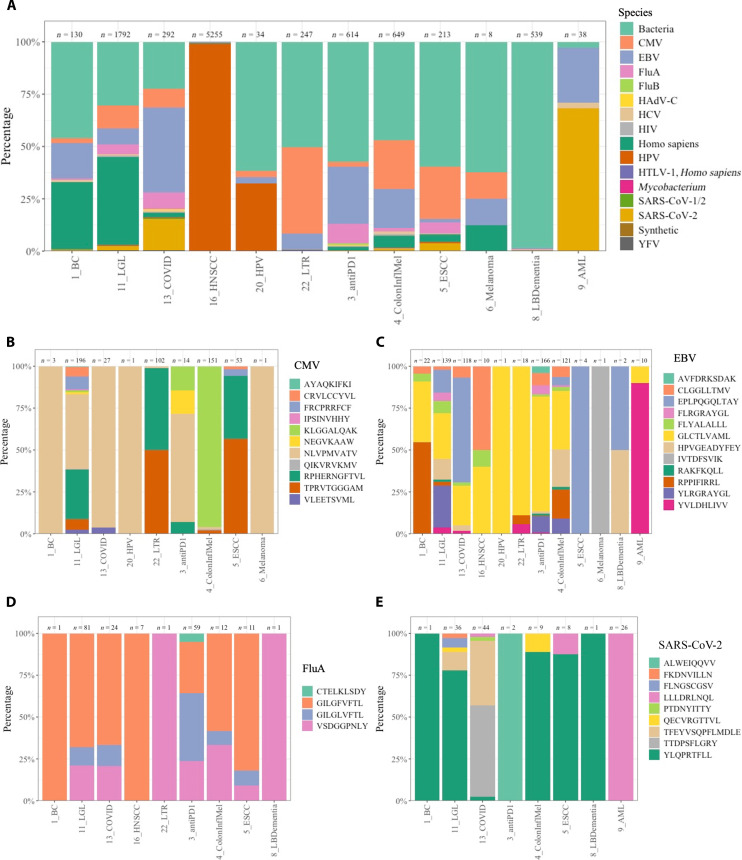
Epitope prediction of the T cell atlas using IMW DETECT. (**A**) Bar graph representing the ~10,000 cells colored by the different infectious species. (**B** to **E**) Individual epitopes for (B) CMV, (C) EBV, (D) FluA, and (E) SAR-CoV2. The numbers about each bar represent the total number of sequences. Flu, influenza; SARS-CoV-2, severe acute respiratory syndrome coronavirus 2; HCV, hepatitis C virus; IMW, ImmuneWatch; YFV, Yellow fever virus.

More challenging populations to identify would be from epitope specific T cells that have variable TCR usage. For this purpose, we used the ImmuneWatch (IMW)–DETECT tool to predict epitope specificity for the entire T cell atlas. Of the 293,847 unique TCRs, 9841 were predicted to be epitope specific (score > 0.2) (Fig. 4A; table S3). We noted that the human papilloma virus (HPV) epitope-reactive TCRs were mostly specific to head and neck squamous cell carcinoma [HNSCC; (*19*): GSE180268]. The GEx signature in the top 30 genes included RANK1, TRIR, DUSP2, CXCL13, HLA-DQA2, ATP5 transcripts, and LINC02446 (fig. S1A). To independently identify the TCR and associated gene signature of interest, we also interrogated the TCR that were HPV epitope-specific and overlapped with the PD-1 sort (section S2 R4). The analysis identified a consensus GEx including CXCL13, LINC02446, RACK1, HLA-DQA2, and DUSP4.

Next, as the predictions included common viral disease with a range of epitopes including cytomegalovirus (CMV; Fig. 4B and fig. S1B), Epstein-Barr virus (EBV; Fig. 4C and fig. S1C), influenza A (FluA; Fig. 4D and fig. S1D), and severe acute respiratory syndrome coronavirus 2 (SARS-CoV-2) (Fig. 4E and fig. S1E). EBV and SARS-CoV-2 did not have specific TCR variable genes associated unlike CMV (TRBV10-1, TRAV19, and TRGV10) or FluA (TRAV12-1). Similar to HNSCC, the SARS-CoV-2 and CMV also had ATP5-related transcripts and RANK1.

### Sequence similarity identifies both colitis- and melanoma-specific clusters

Despite the added benefit of using the TCR to initiate the primary analysis, a common complication is that most TCRs are infrequent and private. In addition, all T cells express at least two distinct TCR chains (commonly αβTCR or γδTCR), which further complicates this analysis. To overcome these challenges, we propose the use of TCR clustering to group those sequences with high similarity into T cell groups. Prior research has shown that high TCR sequence similarity within a population is often indicative of a common epitope response (*25*). In addition, we also use the associated GEx and sequence neighbor enrichment to further prioritize if the cluster is likely to have a unified function.

To demonstrate this approach, we reanalyzed a single-cell dataset of colitis complication post-melanoma treatment as it was the only one with all four TCR chains (αβTCR and γδTCR) (*26*). This dataset composed of eight patients with colitis (C), six patients with noncolitis (NC), and eight healthy controls (CT). For details on the overall TCR structure and common clusters, please see the section S2 R5.

**Fig. 5.**
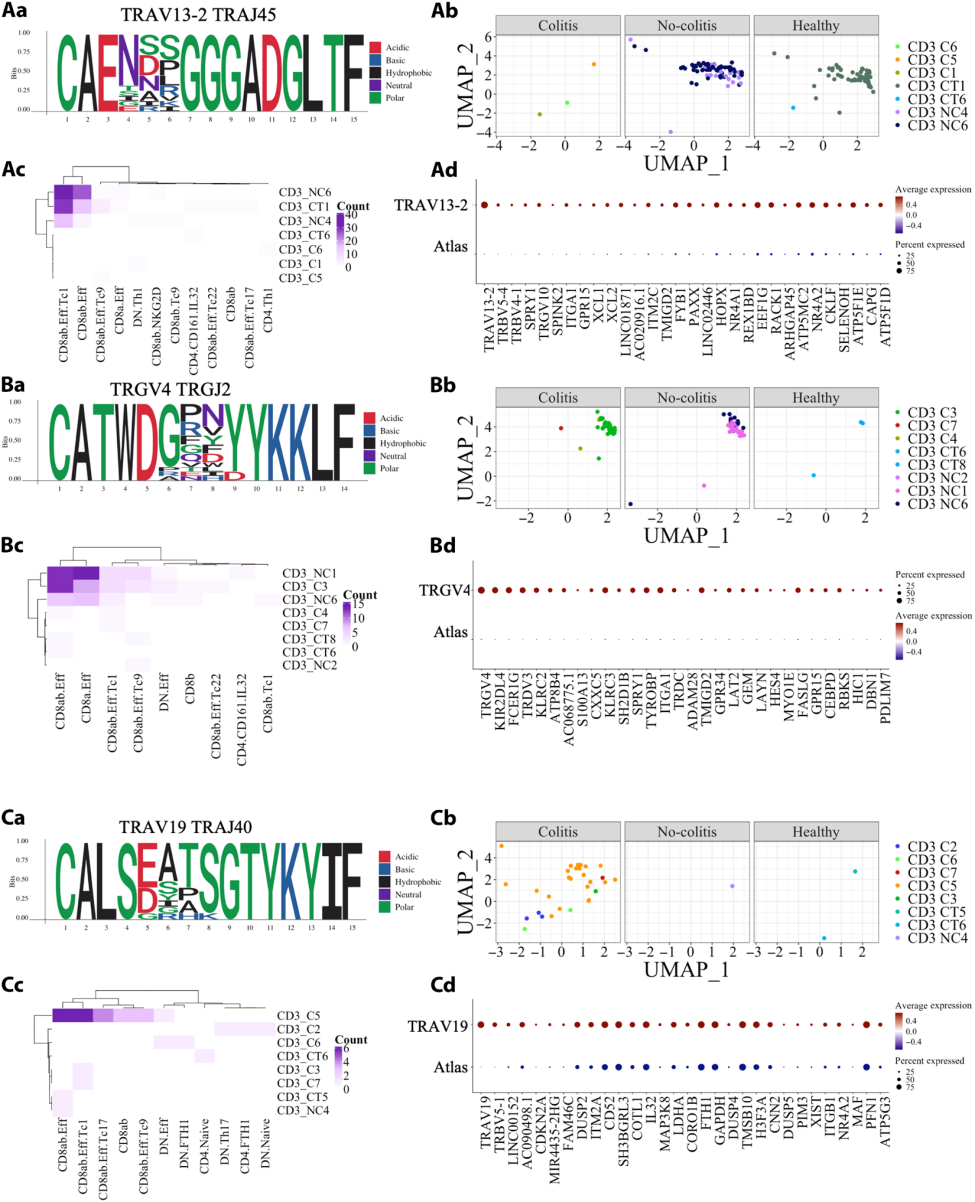
Clustering analysis of the colitis dataset highlighting disease specific clusters. (a) Motif of the cluster, (b) cluster on the UMAP plot colored by individual and split by condition (normal controls, colitis, and no colitis), (c) heatmap of the T cell phenotype versus the individual samples, and (d) the top 30 transcripts enriched on this cluster compared to the remaining atlas (C: colitis; NC: noncolitis; CT: normal controls. (**A**) Cluster 6 (TRAV13-2 TRAJ45) more common in NC and TC. (**B**) Cluster 8 (TRGV4 TRGJ2) more common in melanoma cases. (**C**) Cluster 9 (TRAV29/DV5 TRAJ40) more common in colitis cases.

Interrogation of the α, β, γ, and δ clusters revealed several intriguing patterns (tables S4 and S5). One TRAV13-2-TRAJ45 cluster was present in all three conditions, with more sequences featuring in the CT and NC that had a mixture of effector CD8ab^+^ T cells as well as activation and cytotoxic features (Fig. 5A). Next, we were interested to see whether these cluster may or may not result as a product of high probability recombination events. To this end, we performed TCR sequence neighbor enrichment analysis using clustcrdist. Ten of the 13 unique TRAV13-2-TRAJ45 sequences had significantly more sequence neighbors than expected (TCRdist >12.5 and *P*-adj < 0.05; table S6). These TCR paired with either TRBV4-1 or TRBV5-4. As these cells contained TCR pairings of TRBV4-1 and TRAV13-2, it is possible that this cluster of TCR was CD1b restricted (*27*).

A TRGV4 γ cluster associated with the C and NC cases consisted of CD8ab^+^ γδ T cells with cytotoxic/activation status (Fig. 5B). Again, we performed neighbor enrichment analysis and found that 8 of the 16 unique TRGV4 sequences in this cluster had significantly more sequence neighbors than expected (TCRdist >12.5 and *P*-adj < 0.05; table S6). As these TCRs had minimal presence in the healthy controls as well as being overrepresented and expanded in the melanoma cases (C and NC) thus indicates that they are probably melanoma-associated TCRs.

We also identify an α cluster with TRAV19-TRAJ40 that was overrepresented in the colitis cases (Fig. 5C). Within in this cluster, three TCR sequences had more neighbors than expected and were associated with the CD8^+^ effector phenotype (*P*-adj < 0.05; table S6). This indicated that part of this TRAV19-TRAJ40 cluster may be colitis specific.

The TCR β chains contained fewer clusters of interest, which were mostly specific to a singular individual C3 (fig. S2). The 11-nucleotide oligomer TRBV6-2 (cluster 684) had four clones with significantly more neighbors than expected in the cluster, while the other 11-nucleotide oligomer (cluster 377) and 12-nucleotide oligomer (cluster 343) did not (table S7). Thus, only a few clones in one of the TRBV6-2 may have been colitis specific or could have related to unknown infection. Thus, our TCR-centered approach was able to identify disease-specific αβTCR clusters and γδTCR clusters that were not deducible from a GEx-centered methodology.

### Global TCR motifs associate with invariant T cells and high-generation probability

One of the benefits of combining the 12 datasets was to be able to identify common public patterns (*18*–*20*, *23*, *26*, *28*–*36*). We describe some of the annotation differences observed in the dataset in section S2 R6 and the limited public clone interrogation in section S2 R7. This below section focuses on public clusters common to all 12 datasets. There were three α clusters and three β clusters common to all 12 datasets.

**Fig. 6.**
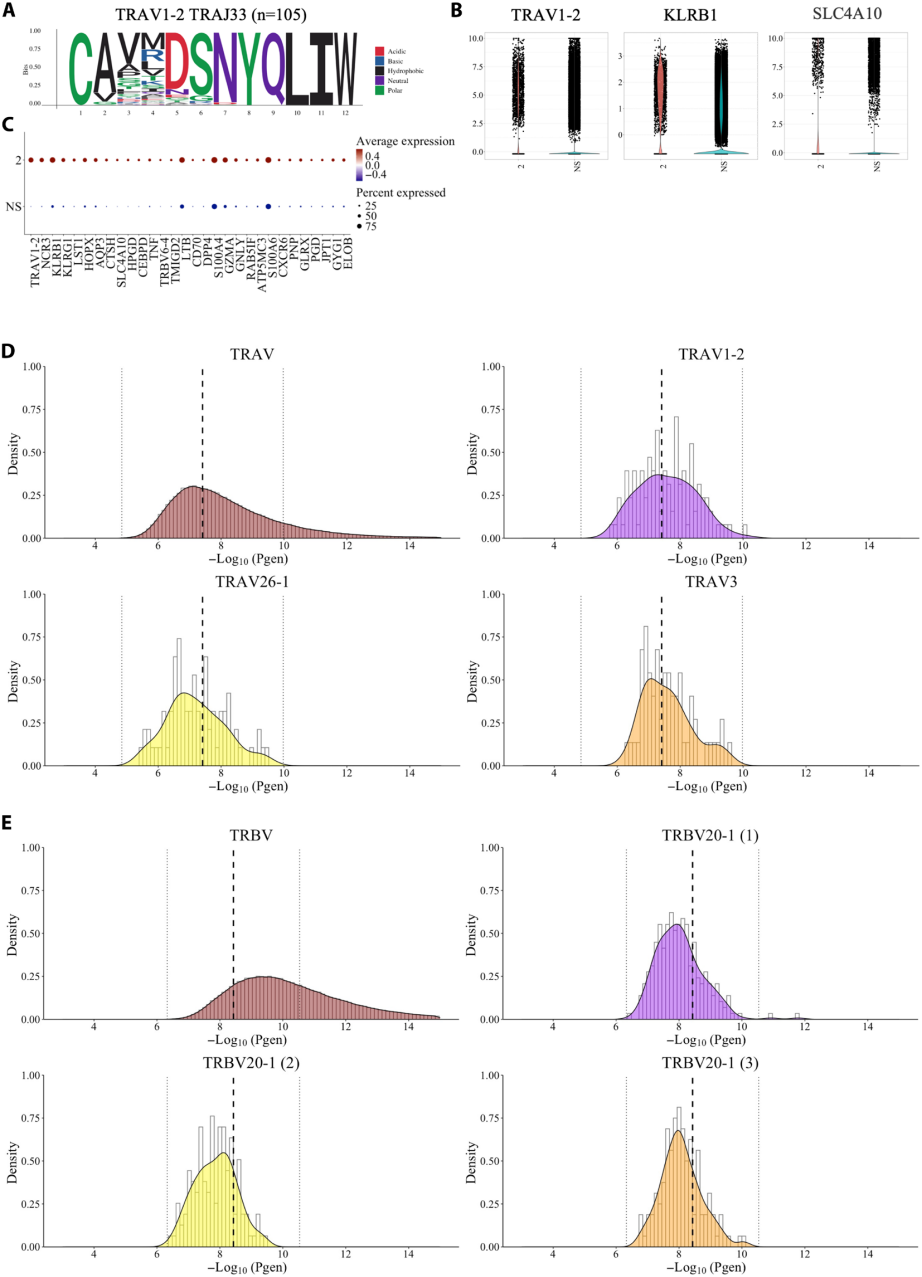
Global clustering analysis TRAV and TRBV clusters present in all 12 samples that had high generation probabilities. The top associated α cluster had the (**A** to **C**) TRAV1-2 TRAJ33 arrangement. This cluster was present as (A) a 12-nucleotide oligomer and (B) corresponding dot plot of the average relative expression that included the expected MAIT-associated genes (C) TRAV1-2, KLRB1, and SLC4A10. In addition, we calculated the probability of generation with OLGA for (D) all TRAV probability of generation curve of the all sequences TRAV1-2 TRAJ33, TRAV8-3, and TRAV27 and for (E) TRBV probability of generation curve of all sequences and the three TRBV20-1 clusters. (**D** and **E**) Middle dashed black line represents the geometric mean of the unique sequences, and the two dotted line represents one SD from the geometric mean. n.s., nonsignificant.

The top α cluster had a junctional length of 12 amino acids and TRAV1-2 TRAJ33 gene arrangement and was present in 99 of the 149 samples. This cluster had 1033 unique paired sequences with 1975 total clones. The DETECT tool predicted 67% (694 of 1033) of the paired unique sequences (score > 0.20) were likely to interact with MR1:5-OP-RU and indicated that the most of these clusters were MAIT (Fig. 6Aa and table S8). When examining the average GEx profile of this cluster, not all cells expressed the commonly accepted MAIT markers, namely, TRAV1-2 along with KLRB1 and SLC4A10 (Fig. 6A, a and c). The two remaining α clusters, TRAV26-1 and TRAV3 were not functionally distinct from the background data. In addition, as the significant genes associated with either were the V gene and generic T cell markers CD44 and CD28 (limited consistent enrichment), it suggests that there may be a diversity of functions for this cluster (fig. S3, A and B). The IMW-DETECT did not identify any epitopes (>0.2) present in either cluster.

Of these TRBV20-1 sequences, in the IMW-DETECT epitope prediction, only three paired TCRs were predicted to be MAIT cells (MR1:5-OP-RU; TRAV1-2 and TRBV20-1) and one TDLGQNLLY (human mastadenovirus C; TRAV27 and TRBV20-1). This indicated that most cells had unknown epitope targets. The three TRBV20-1 clusters had limited genes associated. All three β clusters contained TRBV20-1, had a junction length of 13, and lacked diversity gene inserts (Fig. 6, C to E). Fos proto-oncogene, AP-1 transcription factor subunit (FOS) expression was noted in all three clusters, and this marker is associated with naïve cells (*37*). TRBV20-1 was the most common v_gene, identified ~17.1% of the total dataset (*38*).

Next, we evaluated these six clusters recombination probabilities using optimized likelihood estimate of immunoglobulin amino-acid sequences (OLGA) (*38*). All cluster TCR arrangements were within one SD of the geometric mean relative to the entire dataset (fig. S3, B and C). Therefore, these sequences had a high-generation probability, which may explain their prevalence across the different individuals and disease setting.

Last, we checked to see if any of the TCR sequences was more enriched in clusters than expected (TCRdist > 12.5 and *P*-adj < 0.05). The outcomes are summarized in Table 1**,** and all data are present in (table S9). The 82% of the TRAV1-2 TRAJ33 MAIT, 51% of the TRAV3, and 41% of TRAV26-1 unique sequences in the cluster had neighbor enrichment, indicating that these clusters did not occur by random chance. On the other hand, few sequences in the TRBV20-1 clusters had more neighbor enrichment than expected (2.9, 4.6, and 1.5%). Given the overall TRBV20-1 profiles with few associated differential transcripts and lack of neighbor enrichment, these T cells are probably the result of random chance.

**Table 1.** Summary table of neighbor enrichment for the six common clusters across the 12 studies.

Cluster	Unique sequences in cluster	Unique sequence found in enrichment and cluster	Significant enrichment (*P*-adj < 0.05)
**TRAV1-2 TRAJ33 (MAIT)**	105	90	87
**TRAV26-1**	78	68	32
**TRAV3**	61	48	31
**TRBV20-1 (1)**	239	152	7
**TRBV20-1 (2)**	130	85	6
**TRBV20-1 (3)**	132	78	2

## DISCUSSION

T cells are a complex cell type to interrogate due to their two-dimensional diversity in both the GEx and TCR. Under the current paradigm, GEx information is conventionally used to identify cell-type clusters, which are subsequently labeled with features of the TCR. To shift the process to a TCR-first approach required the development of the tool STEGO.R to uncover actionable TCR-specific features. Here, we re-interrogated and integrated 12 publicly available T cell datasets into a single cell–based T cell atlas, and investigated the need for sufficient background data to ensure enrichment of signal. This atlas can be freely integrated into studies to aid in T cell annotation and to improve enrichment of signals, especially for rarer population/clone, even when TCR data are unavailable (section S3). Overall, the TCR-focused approach was able to identify patterns within the single-cell data that were previously not identified from a GEx-centralized approach.

We first focused on validating the utility of the semi-supervised based annotations. As during thymus development mostly restrict to either CD4 and CD8 that determines future epitope recognition, i.e., CD8^+^ T cells and class I MHC or CD4^+^ and class II MHC (*39*). The vast majority of the top 50 most expanded clones identified were likely CD8^+^ T cells. When examining the unsupervised clustering, many of the clones spanned multiple clusters, leading to lower confidence of the cell’s major CD8 versus CD4 expression markers. The semi-supervised method had greater accuracy especially for the CD8^+^ cells (>95% expression in 43 of the 50 cells). In addition, it appears that the unsupervised clustering often misses small perturbations that are important for the T cell phenotype and necessitates the presence of other T cells of different phenotypes (section S3). While no modeling is perfect, the semi-supervised annotation strategy had greater robustness over the standard unsupervised clustering for T cells. Consequently, as cluster-based process does not represent the T cell biology, utilization of methods that rely on these embedding, e.g., pseudotime in Monocle 3 (*40*), will not be meaningful (section S3). Overall, we recommend adopting this semi-supervised annotation strategy for future T cells studies.

Nevertheless, using global annotation strategies may be limiting understanding of specific T cell clones. We theorized that a lack of detectible difference was due to small GEx subtleties that drive the T cell phenotype that is not captured in PCs used to create the Uniform Manifold Approximation and Projection (UMAP) plot for visualization. To better characterize the dynamic nature of T cells, one can focus on specific TCR clone(s) across multiple time points. In some instances, there may be insufficient clone counts (*n* < 3) either before or after therapy, which was the case in LTR dataset. To overcome this challenge, we compared two of the clones that were more specific to either ACR or treatment. The ACR-specific TCR identified MTRNR2L12, TRGV10, and BIRC3. BIRC3 codes for cellular inhibitor of apoptosis 2 protein that has critical role in CD8^+^ T cell survival (*41*), while less is known about both MTRNR2L12 and TRGV10 as they are pseudogenes. In the LGL T cell cancer dataset, we also identified MTRNR2L12 associated after anti-CD52 therapy (section S3). A recent single-cell dataset identified that MTRNR2L12 long noncoding RNA has an emerging role in CD8^+^ biology (*34*). Therefore, to capture the overall profile, it was necessary to interrogate expanded TCR(s) from two time points or pool TCR compared to T cell atlas as the background to identify a panel of markers associated with the TCR of interest.

Last, we observe significant deficiencies in the definition of GEx-based functional annotation for T cells. A notable example for inadequate annotation protocols in T cell subtypes rely solely on the GEx information level, e.g., MAIT cells identified by TRAV1-2 and SLC4A10 or KLRB1 (*42*, *43*). The MAIT cells were grossly underestimated in the cluster-based approach in the T cell Atlas with much more confidence capture with the TCR-seq that could use the required VJ combination. Another T cell population that we found required the that TCR-seq was the γδTCR, as they could have similar profiles to the adaptive CD8ab^+^ αβTCR (section S3). There emerging evidence that CD8ab^+^ γδTCR have the potential for peptide restriction (*44*, *45*), and they should not be ignored based on outdated understanding of γδ T cells being only innate-like (section S3). The most difficult populations to identify are those with potential epitope specificity as they had hypervariable TCR usage. By using the IMW-DETECT and the tetramer-sorted HNSCC datasets on HPV-specific epitopes, we were able to identify similar transcriptional signature. Overall, the TCR-seq was needed to have higher confidence in finding predication, MAIT, and γδTCR populations. We strongly suggest future single-cell T cell research to include both GEx and TCR-seq with all four T cell chains as this will further improve the accuracy in defining T cell subpopulations.

The full study TRB cluster interrogation identified common TRBV20-1 clusters with various J genes and was also the most prevalent TRBV gene identified. Our analysis identified that these sequences had a high-generation probability and limited transcriptional enrichment with some naïve T cell markers, and most of the cluster had the expected number of neighbors, of which <5% were significantly more enriched than expected. There were also no consistent IMW-DETECT epitopes identified. In the literature, TRBV20-1 TCRs are often among the most common TRBV gene arrangement in a multitude disease settings including COVID-19 (*46*), non–small cell lung cancer (*47*), pancreatic cancer (*48*), rheumatoid arthritis (*49*), and range of cancers and tissue compartments (*50*). However, as the T cell atlas shows, these are highly frequent TCRs across a multitude of conditions due to high-generation probability. Thus, when these TRBV20-1 are found present or even overrepresented within a set of samples or a specific disease setting, this may solely be a proxy for a lack of response as they reflect the naive repertoire or could be a statistical artifact based on the high abundance of TCRV20-1 TCRs and the intrinsic high TCR sample variability. For future studies, caution is recommended before concluding if these common high-generation probability sequences are indeed disease specific. We recommend applying the following strategy: clonal expansion and associated GEx, neighbor enrichment, screening epitope specificity from public databases [VDJdb (*51*)], or using annotation algorithms [TCRex (*6*) or IMW-DETECT].

We highlight through our interrogation that the generic GEx-centric approach is not necessarily the most appropriate methodology for T cell single-cell data, as correct understanding requires the dual modalities. This interrogation importantly showcased the improved robustness of semi-supervised–based annotations that better matched the T cell biology. Collectively, our analyses illustrate that by shifting the interrogation to the TCR repertoire, one can uncover insights into defining T cell subtypes and the identification of disease-associated TCR patterns. Exploring the clustering with TCR sequence neighbor enrichment and epitope prediction modeling could aid in identifying TCR of interest with functional understanding gained from the associated GEx. Moving forward, we recommend a move toward a TCR-first methodology that is facilitated by our STEGO.R application. We believe that this paradigm shift to a TCR repertoire–focused approach will enhance our understanding of T cell biology and push forward therapeutic opportunities.

## METHODS

### Public datasets availability

We selected 22 publicly available single-cell RNA sequencing with single-cell TCR-seq for benchmarking based on a literature search, of which only 12 of the 22 datasets could be processed (table S1) (*18*–*20*, *23*, *26*, *28*–*36*). The main issues for not being able to process the remaining 10 datasets were due to missing information in the public repositories [i.e., no available GEx (*n* = 2) (*52*, *53*), no TCR-seq (*n* = 2) (*54*, *55*)] and data format issue [e.g., summarized TCR data (*n* = 1) (*56*), cannot separate cases in merged files (*n* = 2) (*57*, *58*), and incompatible format (*n* = 3) (*59*–*61*)]. The 10X Genomics data was formatted as either raw filtered files (barcode, features, and matrix) or .h5 file. STEGO.R can currently process the raw files or .h5 standard formats. There is currently no process to extract a usable file from the cloupe and/or vloupe and therefore were not analyzable with STEGO.R. The filtered outputs of the barcode, features, and matrix with the filtered_contig were the most accessible to processing in STEGO.R.

### STEGO.R QC process

The 12 datasets were processed in STEGO.R following the workflow outlined in https://stegor.readthedocs.io/en/latest/ and made use of the organized directory folder setup. Briefly, each of the 150 individual file formats for the quality control (QC) processes was standardized in STEGO.R (step 1). This included formatting the matrix and meta-data with the filtered TCR-seq and downloading the ClusTCR2, TCRex, and TCR_Explore files. As the current 10X Genomics kit and pipeline do not include all four chains, manual processing of the TCR-seq meta data was required for the colitis complication to melanoma treatment dataset (*26*) to merge the αβ and γδ TCR-seq files. The TCRex and ClusTCR2 files were processed in step 2. Each of the 150 matrix files underwent individual quality assessment using the standard Seurat process, and the processed TCR-seq was added to the meta-data, with LGL-healthy6 failing the QC process (step 3a; *n* = 149). These files were then merged using the command-line function (step 3b). To ensure that all cells could be annotated, the files were reduced to the 5006 transcripts and restricted to the TCR-seq that is based on the original combining of the datasets during the merging process. The files were annotated using scGate, annotating 62,000 per loop (eight iterations). The annotations included the T cell functions (table S10) and major, immune checkpoint (IC), senescence, T_H_1 cytokines, and cycling. In addition, TCR-seq information was used to define MAIT cells (TRAV1-2 J33/J20/12), possible CD1b/c restricted, γδ T cells, and remaining αβ T cells (step 3c). Step 3d was used to remove specific cells from the file based on the meta-data. Steps 1, 2b, 3a, 3b, and 3c have command-line scripts available. More details on the inner workings of STEGO.R can be found in section S1.

#### IMW-DETECT

IMW-DETECT (version 1.0) tool (2024; available at www.immunewatch.com/detect) is a user-friendly program designed to facilitate the prediction of epitope-TCR binding for paired TCR. It starts from a TCR file containing a list of paired TCR α and β sequences, which must include information on the V and J gene and CDR3 amino acid sequence. IMW-DETECT annotates every TCR sequence for the most likely epitope target. More information on how to use this tool and interpret the results can be found at https://immunewatch.gitlab.io/detect-docs/.

#### ClusTCR2

ClusTCR2 is an R alternative to the python package ClusTCR (*2*), specifically developed to be applied to single cell TCR-seq data. The original version of ClusTCR applies a two-step process to cluster large sets of TCR sequences. The first step involves encoding TCRs into a numerical vector based on the physicochemical properties of the amino acids. *K*-means clustering is applied to the amino acid vectors to create large families of preclusters. During the second step, a hashing function is used to identify all pairs of CDR3 amino acid sequences with ≤1 Hamming distance mismatch. From these CDR3 pairs, a graph is constructed, and communities are detected using the Markov clustering algorithm, which will be the final clusters.

Because ClusTCR2 was developed with the focus on smaller, single-cell datasets, the second step is sufficient and achieves more accurate clustering results compared to the two-step method. Therefore, ClusTCR2 excludes the amino acid encoding and *K*-means clustering step. As the meta-data, the α and γ (_AG) chains can pair with their respective β or δ chain (_BD). As we used the v_gene, a γ chain will never be included in an α cluster. In the analysis section, we also have separated the α (A), γ (G), β (B), and δ (D).

### Analysis process

These 12 distinct conditions with 149 unique files from 90 individuals contained 493,784 cells having both the GEx and TCR-seq. We aimed to determine if the analyzing TCR-first approach would identify novel patterns that are not discoverable from a GEx-centric approach.

The analysis process was from the perspective of a TCR-seq first approach, while the GEx-centric approach was documented in the original publications and limited to the scRepertoire clonal expansion onto the UMAP with very limited interrogation of the TCR-seq data. The TCR-first approach was able to summarize the total clone count (public versus private) and sequence similarity, where applicable, predicted in TCRex or IMW-DETECT. Each of these sections could identify the overall signature from the scGate annotations and the transcriptional signature expression (overrepresented in TCR/cluster/population) compared to the remaining cells within the Seurat object. The transcriptional signature was found with the “FindMarkers” function in the Seurat Package and visualized as a dot plot (usually restricted to the top 30 transcripts based on statistical significance). The default threshold used was a log fold change of 0.25 and *P* value < 0.05.

To speed up the process, we used the automated function to extract the summary files and corresponding functions. We used either excel or R code to filer the files to identify which of the TCR, clusters, or epitopes to specifically interrogate. Therefore, we did not have to manually create every file and, if needed, update the formatting in the STEGO.R applications for high-quality publication ready files.

Last, we quantified sequence neighbor enrichment through comparison with a baseline model that estimates the expected neighbor distribution in the repertoire. Briefly, TCR sequences are shuffled at the level of the junctions, and a background dataset is constructed from the shuffled sequences. Sequences are selected in such a way that the background approximates the V gene frequency, CDR3 length, and generation probability distribution of the input repertoire. Here, we used a 50× (relative to the size of the input repertoire) background to accurately estimate expected neighbor counts. Sequence neighbor enrichment is calculated for each clone using the hypergeometric distribution. Bonferroni correction was applied to account for multiple hypothesis testing. The thresholds we used for a distance <12.5 and *P*-adj of 0.05 to be deemed statistically significant. For interpretation, those with significant neighbor enrichment indicate that the clustering did not occur by random chance, while the null hypothesis is that >0.05 indicates that the cluster occurred by random change and has lower weight for biological importance. This analysis was performed using the clustcrdist package in python.

## Supplementary Materials

### The PDF file includes:

Figs. S1 to S12 Sections S1 to S3 Tables S1 to S21 References
