# Supplementary material for: T cell receptor–centric perspective to multimodal single-cell data analysis
Source: Sci Adv. 2024 Nov 29;10(48):eadr3196. doi: 10.1126/sciadv.adr3196 (PMC11606500; doi:10.1126/sciadv.adr3196)
Supplement: Supplementary file 1 — Figs. S1 to S12 Sections S1 to S3 Tables S1 to S21 References [file sciadv.adr3196_sm.pdf]

Science Advances
AAAS

Supplementary Materials for

T cell receptor–centric perspective to multimodal single-cell data analysis

Kerry A. Mullan *et al.*

Corresponding author: Pieter Meysman, pieter.meysman@uantwerpen.be;
Kerry A. Mullan, kerry.a.mullan@gmail.com

Sci. Adv. **10**, eadr3196 (2024)
DOI: 10.1126/sciadv.adr3196

The PDF file includes:

Figs. S1 to S12
Sections S1 to S3
Tables S20 and S21
Legends for tables S1 to S19
References

Other Supplementary Material for this manuscript includes the following:

Tables S1 to S19

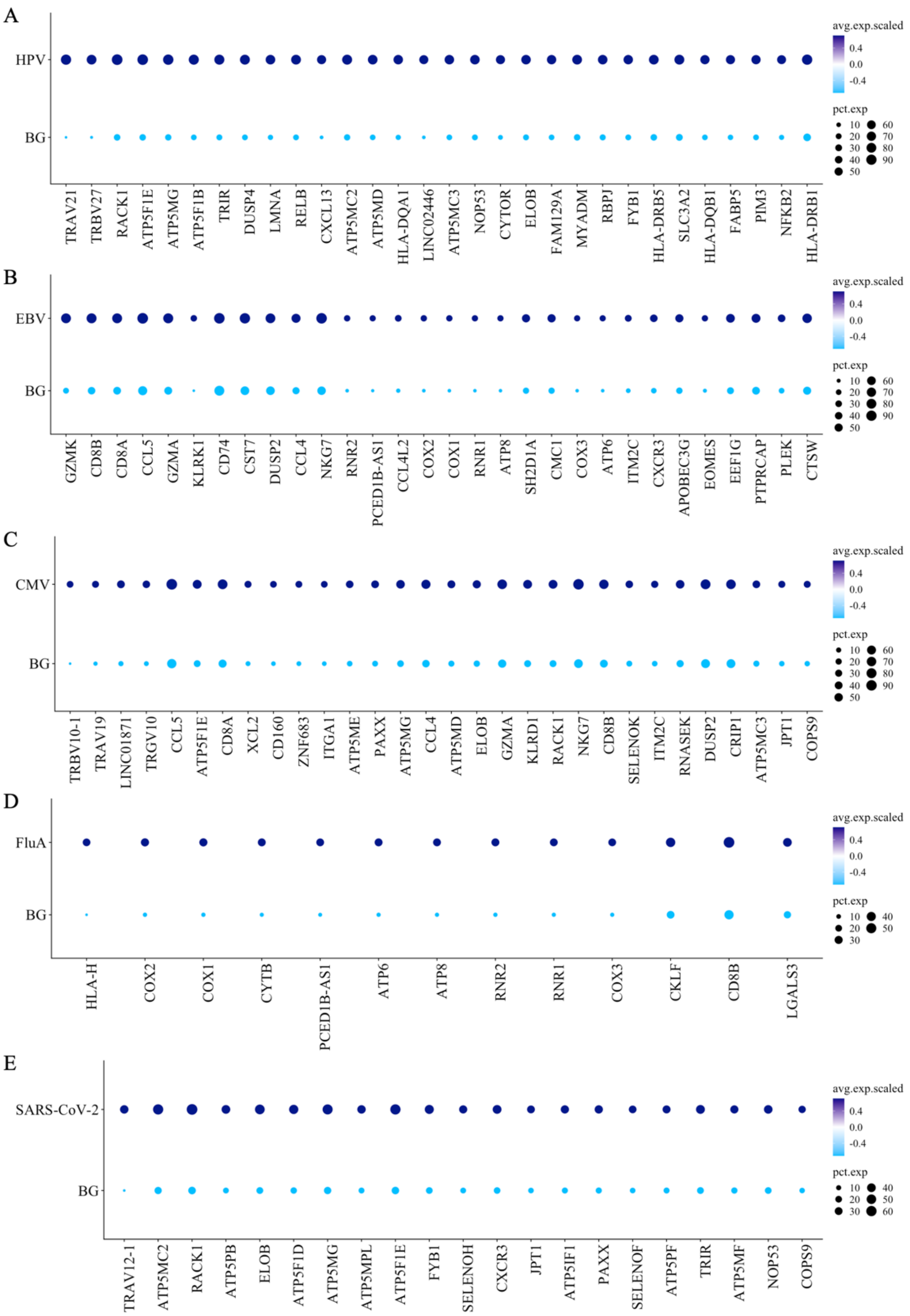

Supplementary Figure S1. Gene expression of the various diseases. The IMW-DETECT epitope predictions for T cells to annotated with (**A**) Human **papillomavirus** (HPV), (**B**) Epstein-Barr virus (EBV), (**C**) Cytomegalovirus (CMV), (**D**) Influenza A (FluA) and (**E**) Severe acute respiratory syndrome coronavirus 2 (SARS-CoV-2). Expression levels shown are those of the T-cell annotated with these viruses, compared to those within the T-cell atlas without. BG, background; BG in this case is the remainder of the T-cell atlas.

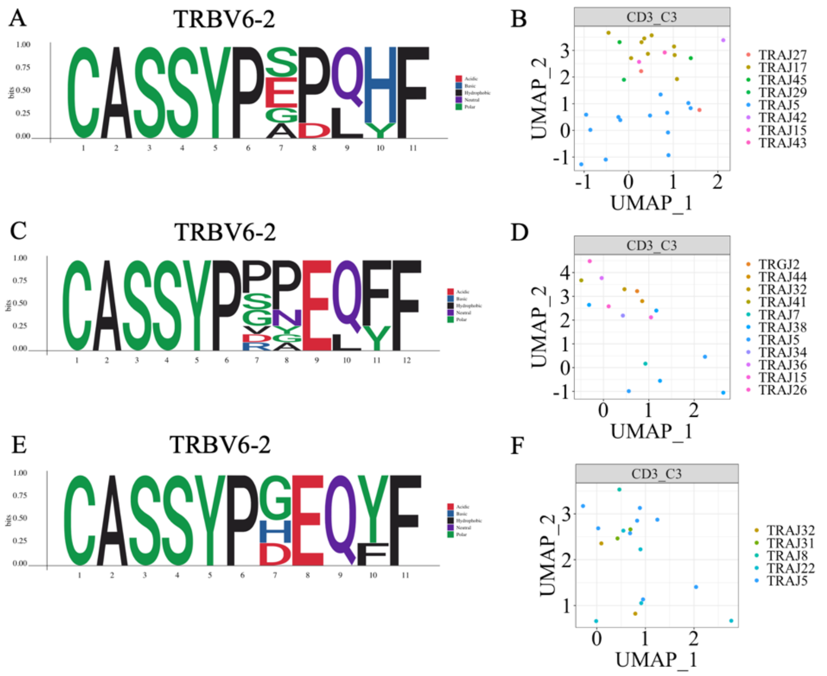

Supplementary Figure S2. C3-colitis dataset showcasing three private clusters. (**A-F**) Three TRBV6-2 clusters from the C3-colitis individual. The three TRBV6-2 (**A,C,E**) motif plot are showcases as two (**A and E**) 11-mers and one (**C**) 12-mer. (**B,D,F**) UMAP plot was split by the individual and colored by TRAJ gene. C = colitis.

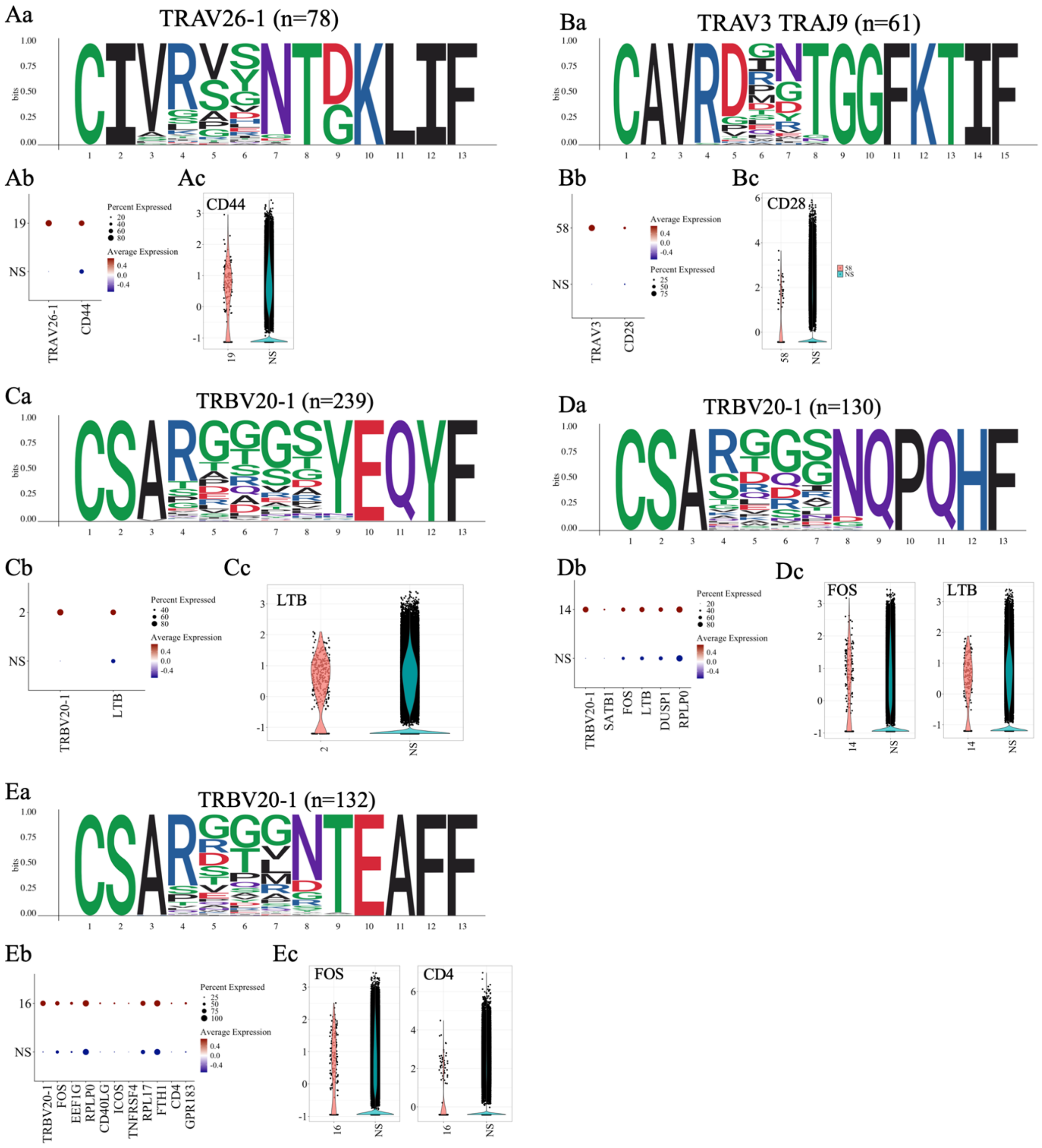

Supplementary Figure S3. Global clustering analysis of two TRAV and three TRBV common clusters. Represent the remaining two (**A**) TRAV8-3 and (**B**) TRAV27 clusters as well as the (**C-E**) three TRBV20-1 clusters. The numbers in the brackets on the (**a**) Motif represent the total number of unique sequences; the number in the brackets represents the number of unique sequences present. (**b**) dot plot and (**c**) violin plot of some of the transcripts associated with the respective cluster.

Section S1

Inner workings of STEGO.R

STEGO.R was created to aid in automate the T cell receptor repertoire analysis and prioritize TCRs in context of the gene expression from single cell experiments.

If a user has already processed the single cell data using R and scRepertoire proceed to step 3e, otherwise, start from step 1.

The program supports outputs of either Cell ranger (10x Genomics) or Seven Bridges (BD Rhapsody) (**Supplementary Table S20**). As there are slightly different formatting between the technologies, they require distinct inputs.

Install STEGO.R.

For details see the https://stegor.readthedocs.io/en/latest/

Running the program.

```
require(STEGO.R)
```

```
runSTEGO() or STEGO.R::runSTEGO()
```

Setting up the project.

Download the Director_for_project that contains the directory structure of each project. Rename the "Director_for_project" folder as desired. For instance, "BC_2024" for the breast cancer dataset. The directory contains several folders that will be used for the processing (**Supplementary Table S21**).

Performing the QC process

STEP 0. Store all the raw file's outputs in folder 0_rawfiles.

STEP 1. Upload the raw files into the QC section depending on the technology. Download each of tab outputs into their respective folders; 1_TCRex (TCRex.tsv), 1_SeuratQC (cell by gene matrix and meta.data file), 1_TCR_Explore (TCR_Explore.csv) and 1_ClusTCR (AG_clusTCR.csv and BD_clusTCR.csv).

Alternatively, this process can also be performed using the function in the **preprocessing.R** file. This process requires that the sub-folders (e.g., Sample_treatment) within the 0_rawfiles, need to contain the barcode, features, matrix and contig file.

STEP 2a. TCRex processing. Merge the TCRex files (if required), and upload (max 50,000 unique sequences) to the TCRex webtool. Download the output and save the **4_Analysis** folder.

STEP 2b. ClusTCR2 processing.
Merge the AG_ separately from the BD_ files. Load the merged file under the clustering inputs tab, click 'run clustering' and wait for ClusTCR2 to run. Download each of the AG_ and BD_ file to **4_Analysis** folder.

STEP 3a. Perform the Seurat QC for each file. Select the cut-off on sample mitochondria genes (mtDNA), ribosomal RNA (rRNA), feature counts. These cut-offs will change depending on

the organism, and differences between focused panels (BD Rhapsody immune panel) and full transcriptome (both technologies). For instance, the default 10x Genomics parameters are <20% mtDNA, >5% rRNA, 200 to 2500 features. Low rRNA are likely sequencing issues. Higher mtDNA identifies cells that are damaged mitochondria and may represent a technical error, rather than disease specific features. Low features represent poor coverage, while higher feature counts likely represent doublets [doi:10.1186/s13059-016-0888-1]. before starting the process, the user adds in their file name, as it will be included in the 'orig.ident' column. The user will then select the number of principal components for dimensionality reduction (default: 15; usual range 10-15), as well as the number of distinct clusters. Once the QC of the Seurat object is finished, the meta-data containing the TCR sequences are added. The files is then saved in the 2_SCobj folder. This step is repeated for all files in your experiment. As there is a need to visualize the cut-off, this QC processing step was not automated. See https://satijalab.org/seurat/ for details on the process.

STEP 3b Merging and batch correction. Merge the multiple Seurat files from the 2_SCobj. This process will restrict the number of features to a maximum of 5036 that was based on the 5000 most variable transcripts of the 12 datasets 5000 and the transcripts required for annotating. This step reduces the overall file size, enabling the ability to merge larger datasets, which may help decreasing computing time for the analysis. Save the ProjectID_merged.rds file in the **2_SCobj** folder. The merged file will then need to undergo batch correction with Harmony. This process follows the Seurat QC: find variable features, scale data, compute principal components, Harmony batch correction and UMAP dimensionality reduction. This batch correction is based on the 'orig.ident' column. Once completed, save the ProjectID_harmony.rds file in the 2_SCobj folder.

Step 3c. Annotating the Seurat object. Upload either the single sample file or merged file to annotate. Select the appropriate model, dependent on sequencing company and library size (e.g., all transcripts vs focused immune panel). These strategies have been modified for the BD Rhapsody Immune panel which has fewer genes and requires a high scGate threshold. Additionally, the current version also adds TCR-seq based annotations and needs to be added separately from the scGate annotation. We recommend the following annotation order: TCR-seq annotations, pre-defined scGate models and custom annotation. The final Seurat_annotated.rds file is saved to the **4_Analysis** folder.

After the refinement process, this should result in five scGate annotation models and a TCR-seq model for humans: T cell functions, major markers, cycling, senescence, immune checkpoint, as well as the TCR-seq based annotations for the invariant T cells e.g., MAIT, iNKT and distinguishing γδTCR and remaining αβTCR.

Custom annotation models:

1. Decide which genes to use for the annotation model.
2. Under the 'marker check' tab you can view if the genes are present via the feature plots and have been scaled. If not present, or lowly present relative to the CD8A expression, use with caution as they may miss many cells, which is the case with CD4 in many experiments.
3. In the master_table.tsv file add in the marker sets under 'name' and the gene ID's under 'signature'. The genes are separated by ';' and each gene is considered as an OR variable in scGate (either can be present).
4. Create the requirements of each annotation (e.g., TcellFunction). The file includes the following headers 'levels', 'use_as', 'name' and 'signature'. Levels column set as 'level1,

level2 … levelx' acts like the gating strategy similar to flow cytometry and is considered as an 'AND' statement and if they are present which is designated as either positive or negative in the 'use_as' column. Update the suffix to the required annotation e.g., Early_scGate_model.tsv. the '_scGate_model.tsv' is required to identify the files to add to the annotation model.
5. Update the name of the annotation model and run the model.
6. Check the locations of the annotations under the "UMAP check" tab.
7. Download the annotated file.

Note: New annotations can be added to an already annotated file, as required.

STEP 3d (optional). Remove unwanted aspects of the file. Even after the data has been process, the user may wish to further clean there file by removing samples based on any of the columns within the meta data table. For instance, in BD-Rhapsody datasets that use multiplexing, which can result in ambiguous origin with either multiplet or undetermined. These identifiers are located under the 'Sample_Name' column. This step can remove other unwanted samples as well (e.g., *NA* from the chain to restrict to both TCR and GEx). Save the file ProjectID_keep.rds to **4_Analysis** folder.

Note: this step can be done before the annotation step.

STEP 3e. converting scRepertoire files into the STEGO.R format. If users have already completed the QC process and have formatted the data in the scRepertoire formatting, this step will convert

STEP 4. Analysis

The analysis folder will contain several files required for interrogating the breadth of the repertoire including the annotated Seurat (.rds) file, clustering outputs (AG_ and BD_ .csv) and the TCRex (.tsv) outputs in the 4_Analysis folder. If required, for more complex experiments, the user can update the Update_ID.csv file, this file will match the ID used under the "Sample_Name" column. The "Sample_Name" column was added to the 10x Genomics pipeline, and is included in the "Sample_Tag.csv" file in the BD Rhapsody pipeline. The user can then change the 'Selected Individual', 'Colour by:' and 'Split graph by:' to the updated values as needed.

The user can upload the .rds file and it will automatically detect if the file was of BD Rhapsody or 10x Genomics origin, as well as the species. The BD Rhapsody detect if numeric were used in the Cell_Index, and the ending of '-1' in the 10x Genomics. The species are detected from the scaled data for the presence of uppercase letters for the first three characters (*Homo sapiens* nomenclature) or uppercase first letter followed by lowercase letters (*Mus musculus* nomenclature).

The Analysis section has three main sections: overview, TCR and GEX, and prioritization.

Overview section

The overview section allows visualization of clonal expansion (TCR tab) and the gene expression (GEX tab). Unlike other programs/packaged (e.g., scRepertoire), which take the approach of performing the analysis of gene clusters annotations and then describe the TCR, we did not go into depth on this analysis approach.

The clonal expansion categories are based on the count category definitions described in scRepertoire (*16*): single (n=1), small ($1<x\leq5$), medium ($5<x\leq20$), large ($20<x\leq100$) and Hyperexpanded ($100<x\leq500$) and frequency categories: rare (<0.0001), small ($0.0001<x\leq0.001$), medium ($0.001<x\leq0.01$), large ($0.01<x\leq0.1$) and Hyperexpanded ($0.1<x\leq1$). This can be visualized on either a bar plot or overlayed on the UMAP. Additionally, the program introduces identifying the clonal overlap (downloadable Table) and visualized as an upset plot (<31 groups) .

The GEx tab allows the user to visualize the data on either the UMAP plot or as a proportion in a pie chart.

TCR → GEX

Unlike previous application and processes, the focus of STEGO was to interrogate the TCR repertoire and then identify their correlated gene expression. This section is separated into several sub-sections: Top clonotype, Expanded, ClusTCR2 and Epitope.

Common visualization and analysis for each section: summary table, UMAP of the selected TCR, pie chart of expression of the selected TCR, positive markers associated with the TCR relative the rest of the dataset, dot plot to display of the significant genes, and the over representation (all except Marker). The UMAP plots can be displayed as the overall sample or split by the group comparison "Include group comparison" to 'yes'.

Over representation gene sets: There are 303 gene sets that are from various sources: Gene ontology biological pathways (GO BP), CellTypist, Reactome, KEGG, C8, MSigDB. Additionally, we also added in STEGO.R based gene sets (*see section 2.6.10 for details*).

Top clonotype: Summarizes the data based on the chain information with the default 'vdj_gene_cdr3_AG_BD' which ensures paired chain interrogations and can be changed as required. The default summary table is for the entire dataset, and if needed can be based on a single sample (set by: Display one individual? Yes, and then "Display one individual" to the selected individual). Once these variables have been decided, proceed to the stats tab, followed by the dot plot (can be restricted to number of genes), and the over representation analysis.

Expanded: This defines the expanded (Ex) vs the non-expanded (NEx) T cell clonotypes regardless of their specific sequences. This section can determine if the Ex have distinct signatures across samples, relative to the NEx of one sample. Alternatively, the user can also compare sample specific differences if they input the _Ex and _NEx into the 'Samp 1' and 'Samp 2' column. The default column to summarize is the 'vdj_gene_CDR3_AG_BD'. The user will select the column to include "Sample column name" and select those to include in the 'ID's to include' box. In the side bar panel, the user can set Ex threshold based on frequency of the repertoire from in the "Cut off greater than' column. Alternatively, they can select the minimum # of clonotypes in the "Cut off greater than". The UMAP will display the Ex and NEx clones. Once these variables have been decided, proceed to the stats tab, followed by the dot plot (can be restricted to number of genes), and the over representation analysis.

ClusTCR2: This section focuses on the sequence similarity based on the ClusTCR2 package, which is the R based version of ClusTCR (*2*). The user will upload both AG_ and BD_ files separately. The side bar will contain two variables that the user to alter which cluster is displayed: "Clusters to display" (numeric value) and "Chain to display" (AG or BD). The order of the clusters is based on total number of nodes per cluster. The cluster motif can be visualized

under the ‘motif’ tab which also displays what the Variable and junction genes association with the sequence and from what samples. Once these variables have been decided, proceed to the stats tab, followed by the dot plot (can be restricted to number of genes), and the over representation analysis.

Epitope: Epitope prediction from TCRex. There are up to 100 epitope models within TCRex based on single Beta chain, TRBV and TRBJ. The TCRex file adds in the epitope and pathology. The user can view the summary file based on three variables: function (Colour Pie by (hm = y-axis)), group (Split Pie by (hm = x-axis)) and individual (Selected Individual). The user can then impute the epitope information, which is in order of highest # of total clones. Once these variables have been decided, proceed to the stats tab, followed by the dot plot (can be restricted to number of genes), and the over representation analysis.

Marker: Identify TCR’s associated with a single or dual markers. This section uses the scaled data for the visualization and analysis. The single marker section was used to identify which TCR’s are associated with markers of interest. Set the threshold of ‘Marker +ve cut-off (>)’ by interrogating the violin plot that is separated by “Sample_Name” (**Suppl. Fig. S4A**). If the violin plot looks like **Suppl. Fig. S4B**, it is likely to be a technical issue rather than a true result. We noted that many of the cytokines had this pattern and were unlikely to be real, and therefore were removed from the annotation models. Once these variables have been decided, proceed to the stats tab, followed by the dot plot that can be restricted to number of genes, and the over representation analysis. The dual marker section follows the same process as the single marker section except two thresholds and selecting the quadrant of interest.

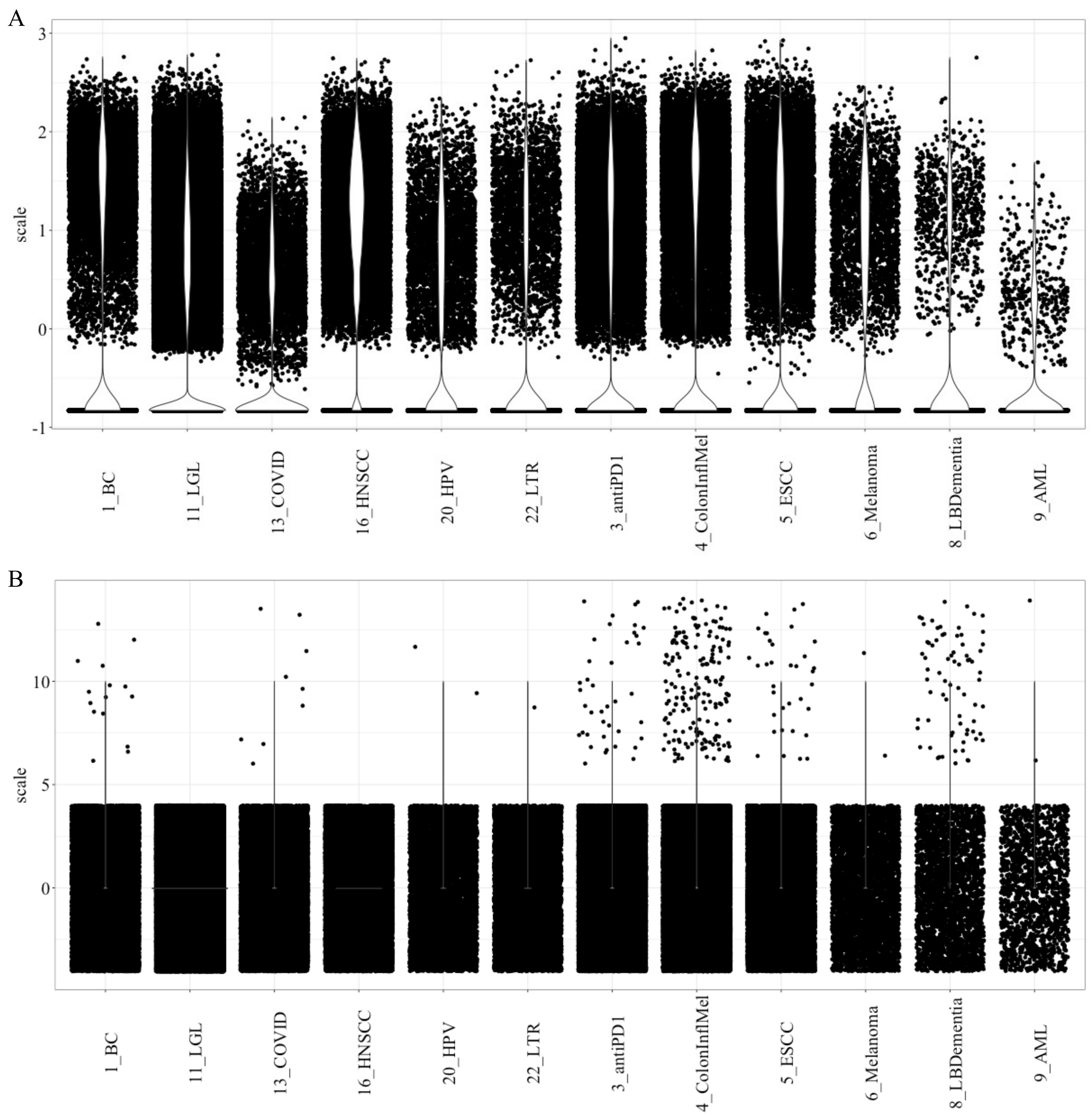

Supplementary Figure S4. Violine plots showcasing quality and poor transcript expression. (**A**) high quality expression of CD8A, while (**B**) technical error of IL4. The x-axis of the violin plot represents the unique studies, and the y-axis is the scaling of the data.

Automating and TCR prioritization.

The automated strategy is found under the 'prioritization' tab. This section was split into three sub-sections: clonotype, cluster and epitope/annotation. The automated approach will use a minimum of three (>2) cut-off due to statistical requirement of the minimum sample size of three.

<u>Clonotype</u>

This section detects the type of data of single sample or multi sample based on the 'Selected Individual' variable (default: Sample_Name). The single cell dataset the user determines the percentage of the repertoire (side bar panel). The user can also increase the percentage until no 'immunodominant' clones are detected. The program will then compare the expanded vs the non-expanded clones.

When the program detects multiple samples, there is priority value to the clonotypes based on:

1/(total clonal frequency x total number of samples)

Therefore, the order of analysis is based on smaller priority to largest. The data frame is then subset to default to a minimum of two or more samples for the multi-sample analysis. The user can then set the threshold for the multi-sample priority where the summary is displayed in the table. This multi-sample detection section also identifies unique clonotypes (private), and the user can set the threshold (default >2).

The program will download the files in the 'multi → Publiclike' or 'Multi → private' folders respectively in the 'directory_for_project'. The downloaded files include clone summary table and for each clone the find marker statistic table, dot-plot and over-represent table. The multi-sample will also download an upset plot if <31 samples are detected. The program will not download the overrepresentation table if no genes were found.

<u>Cluster</u>

The clustering section will require the user to upload both the AG_ and BD_ clustering file. The program updates based on the calculated priority score. This score is calculated the order based on:

1/(total clonal frequency x number of nodes x total number of samples)

Therefore, clusters with clonal expansion, more connections and present in multiple samples have higher weight. The user can change the threshold for both the separate AG and BD files. The user can update the threshold to decrease the total number of clusters analyzed.

The program will download the files in the 'prioritization → clustering' folder in the 'director_for_project' with output a clustering summary table and per clone will download the motif plot, find marker statistic table, dot-plot and over-represent table.

<u>Epitope/Annotation</u>

The user will upload the TCRex.tsv file that contains the beta and epitopes of interest. We recommend running both the 'pathology' or 'beta' (beta CDR3 sequence) for each epitope.

The function can also be used more broadly to the annotations that prioritizes the data based on a 'group' and 'function' e.g., Sample_Name and TcellFunctions.

The program will download the files in the 'Prioritization → EpitopePred' folder in the 'director_for_project' with output the epitope summary table, heatmap Fig. and per epitope/annotation will download a UMAP plot, find marker statistic table, dot-plot and over-represent table.

GEX → TCR

This section focuses TCR's associated with certain annotations or specific markers (single or dual). As this was the previous approach to analyzing TCR per cluster annotation, this was not the focus on STEGO.R development. The automated extraction can be done under the <u>Epitope/Annotation.</u> For our purposes we used this section to validate which transcripts to use for the annotation modelling.

Section S2

R1 Understanding the dynamic of the malignant T cells of a partial responder to anti-CD52 therapy.

The most hyperexpanded clones originated from the large granular lymphocyte (LGL) leukemia dataset, a T cell cancer (23). The original purpose of the LGL study was to block CD52 and cause apoptosis of the over-proliferated T cells. While some cases fully responded to treatment, the anti-CD52 (alemtuzumab) only caused a partial response in some patients including UPN4. For this analysis we focused on the partial-responder UPN4, with the aim to identify potential reasons for the lack of response.

UPN4's highly expanded clone (n = 36,846), TRAV12-2.TRAJ9 CAATTGGFKTIF & TRBV20-1.TRBD2.TRBJ2-4 CSATEGNIQYF was likely driving their T cell malignancy. The clone featured a predominant CD8+ effector-like phenotype that contained a mixture of phenotypes based on our T cell functional annotation model (**Suppl. Fig. S5A**). Despite the prominent CD8+ phenotype, the standard unsupervised cluster-based annotation labeled several clones as CD4+ in pre: 5.7% and post: 2.6%. In contrast, our improved semi-supervised annotation model identified only pre: 0.43% and post: 0.07% with the CD4+ marker (**Suppl. Table S11**). The semi-supervised method thus had more accurate CD8+ annotations with ~13x (pre: 5.7/0.43) and ~37x (post 2.6/0.07) fewer incorrect CD4+ assignments (**Suppl. Fig. S5B**). To confirm the robustness of the semi-supervised annotation strategies, we analyzed the other top 50 clones with a threshold >95% for the CD8 marker. The semi-supervised approach identified 43 of the top 50 clones as CD8+, while using the unsupervised strategy only six clones met the 95% expression threshold. When annotating these CD8+ clones with the semi-supervised, they had fewer CD4+ miss-calls compared to the unsupervised approach (**Section S2 R2**).

The TRAV12-2/TRBV20-1 clone CDR3 nucleotide sequence with 36,838/36,846 clones had the same nucleotide arrangement, TGTGCCGCGACTACTGGAGGCTTCAAAACTATCTTT and TGCAGTGCTACCGAGGGAAACATTCAGTACTTC, suggesting one originating event. To further characterize the TRAV12-2/TRBV20-1 clone through the measured gene expression of IFNG and TNF. The function of a T-cell is often assessed by the cytokine panel of Th1-based molecules IFNγ (IFNG) and TNF in experimental assays. Based on associated transcript markers, we identified that this T cell clone had a mixture of Th1 cytokine expression with subpopulations either being IFNG+TNF+ (3.8%), IFNG+ (46.3%) or TNF+ (2.6%) (**Suppl. Fig. S5B**). This illustrates how a single T cell clone is potentially differentially responding and depending on which cytokines they co-express and is reflective of what we observed in functional experiments. However, using known markers did not reveal substantive differences from either the population T cell phenotypes or Th1 cytokine expression pre- and post-anti-CD52 therapy that could explain the reason for the partial response.

To understand the impact of the anti-CD52 treatment on this T cell clone, we compared the transcriptional profile across time points (pseudo bulk analysis). The top transcript associated with post-treatment was MTRNR2L12 an emerging pseudogene that appears to have some relevant to CD8+ biology (62) (**Suppl. Fig. S5C**). There were also several transcripts with

lower expression in post-treatment including IL7R, LTB, HSPA1B and CD52; the latter transcript's protein was the intended target of the anti-CD52 treatment (**Suppl. Fig. S5D**). We can thus theorize that the reduced and lower expression of these genes may possibly explain the partial response of UPN4. This insight could only be found in the TCR-first approach as it was missed in the original GEx-centric analysis due to unsupervised global annotations and not interrogating specific clones.

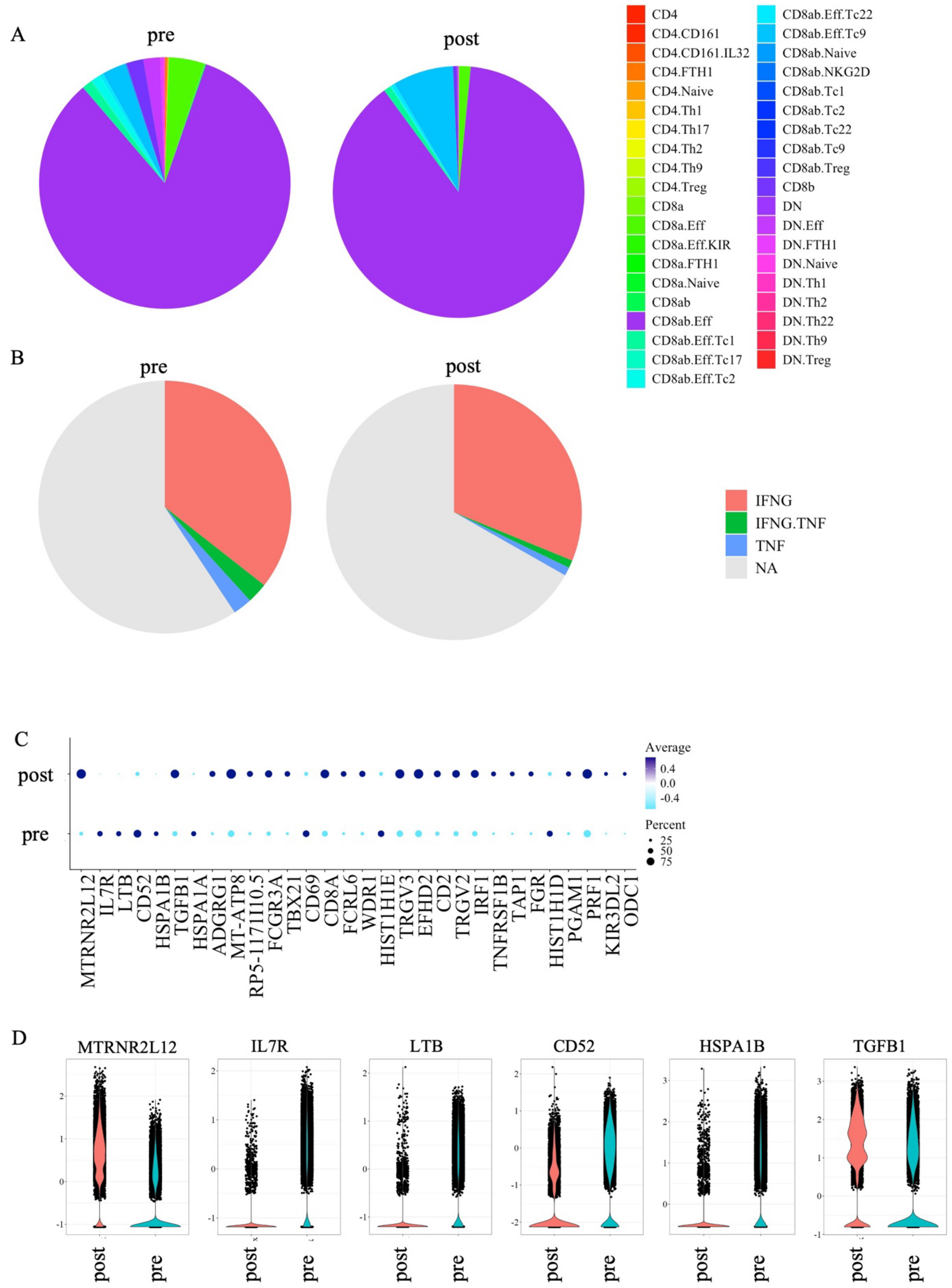

Supplementary Figure S5. TCR-first approach identifies panel of genes post anti-CD52 from the malignant T cell TRAV12-2 & TRB20-1 clone from the partial responder UPN4. TRAV12-2.TRAJ9_CAATTGGFKTIF & TRBV20-1.TRBD2.TRBJ2-4_CSATEGNIQYF representing as (**A-B**) pie chart colored by the scGate modelling of (**A**) T cell functions using and (**B**) Th1_cytokines (IFNG and/or TNF). Comparing the pre- and post-anti-CD52 treatment showed as the (**C**) dot plot of the top 30 markers that were significantly different using the FindMarker statistic, and (**D**) the top seven genes expression represented as the violin plots of the post- and pre-treatment. For the violin plot, each dot represents a unique cell.

R2 Improve annotation strategy with semi-supervised approach.

The common annotation strategy uses the unsupervised annotation approach and requires determining the appropriate number of clusters. This can change with the addition of new data. With this combined dataset we set the resolution to 1 and resulted in 23 clusters. The find marker was used to identify the markers enriched in each cluster relative to everything else. If statements were then used to identify if the CD4 or CD8 marker were enriched for each of the 25 clusters. The feature expression of CD8A and CD4 shows various coverage for each of these major T cell markers within each cluster (**Suppl. Fig. S6**). Therefore, neither CD8A nor CD4 were major contributing feature in the PCs that are used as inputs for the to create the unsupervised clustering.

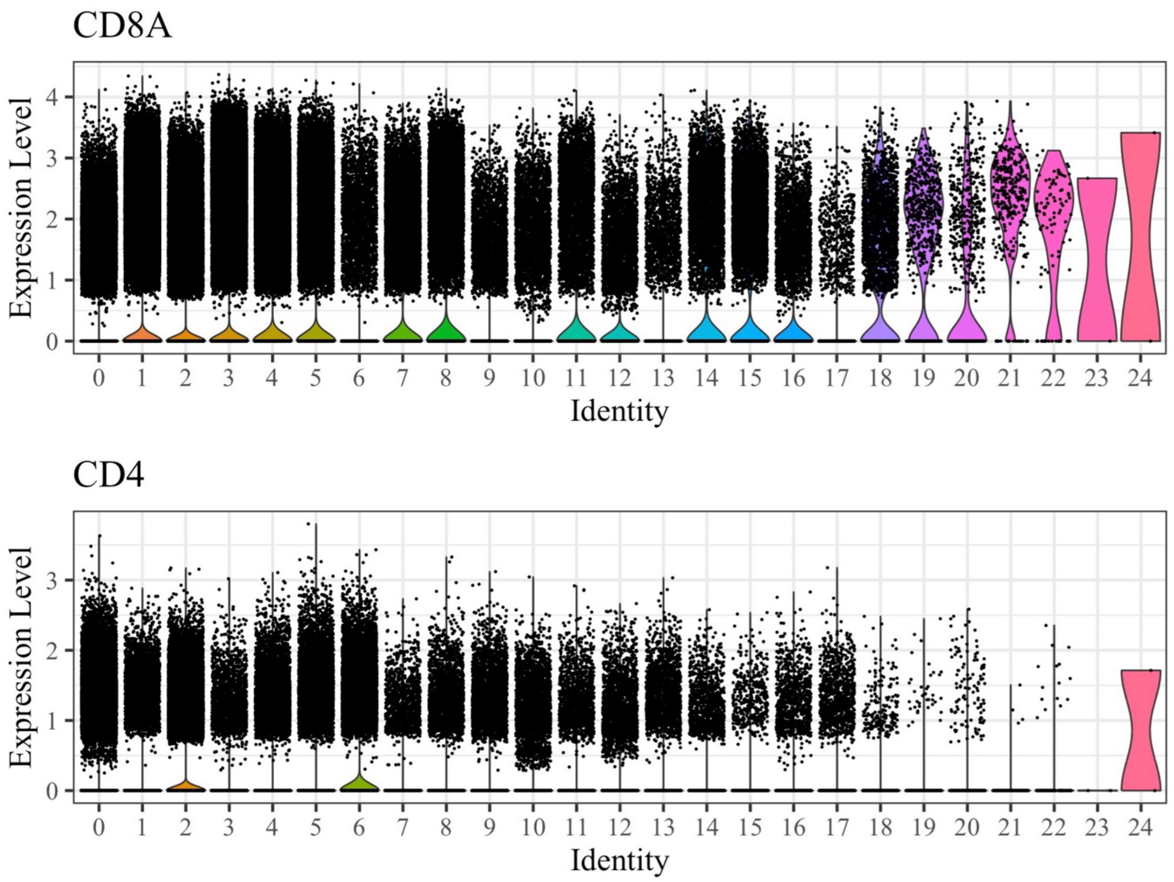

Supplementary Figure S6. Expression of CD8A and CD4 per cluster. The violin plots are split by the 25 clusters (0 to 24) and highlighting the (**top**) CD8A marker and (**bottom**) CD4 from the T cell Atlas ~500k cells. Each dot represents a unique cell. Each plot has the scaled expression from 0 to 5.

To confirm if the semi-supervised annotation strategy was more accurate than the unsupervised clustering, we interrogated the top 50 most expanded clones. For the CD8+ cells, the cluster-based approach identified a mean expression of 75% per clone (six clones with >95% of the total cells/clone expressing CD8) and the semi-supervised a mean expression of 94% (43 clones with >95% of the total cells/clone expressing CD8 (**Suppl. Fig. S7; Suppl. Table S12**). Only one of the top 50 clones appeared to be a CD4+ T cell. This cell only had 50% coverage CD4 annotation in the semi-supervised approach, which it would have been mislabeled in the unsupervised with <1% deemed to be CD4 (**Suppl. Fig. S8**). The lower confidence of the annotations is observed because the clones can span many of the unsupervised clusters and diluting the signal for assigning even the major T cell markers of CD4 and CD8 that determine

function i.e., HLA/MHC specificity of class I or class I peptide presentation. This data identified that CD4 and CD8 were not among the major features to create the UMAP and had more diffuse expression across the clusters, indicating the issue in using the strategy for cell annotation. Overall, validating with the TCR-seq we were able to confirm that the semi-supervised annotations had higher confidence at calling CD8+ T cell assignment then the unsupervised strategy.

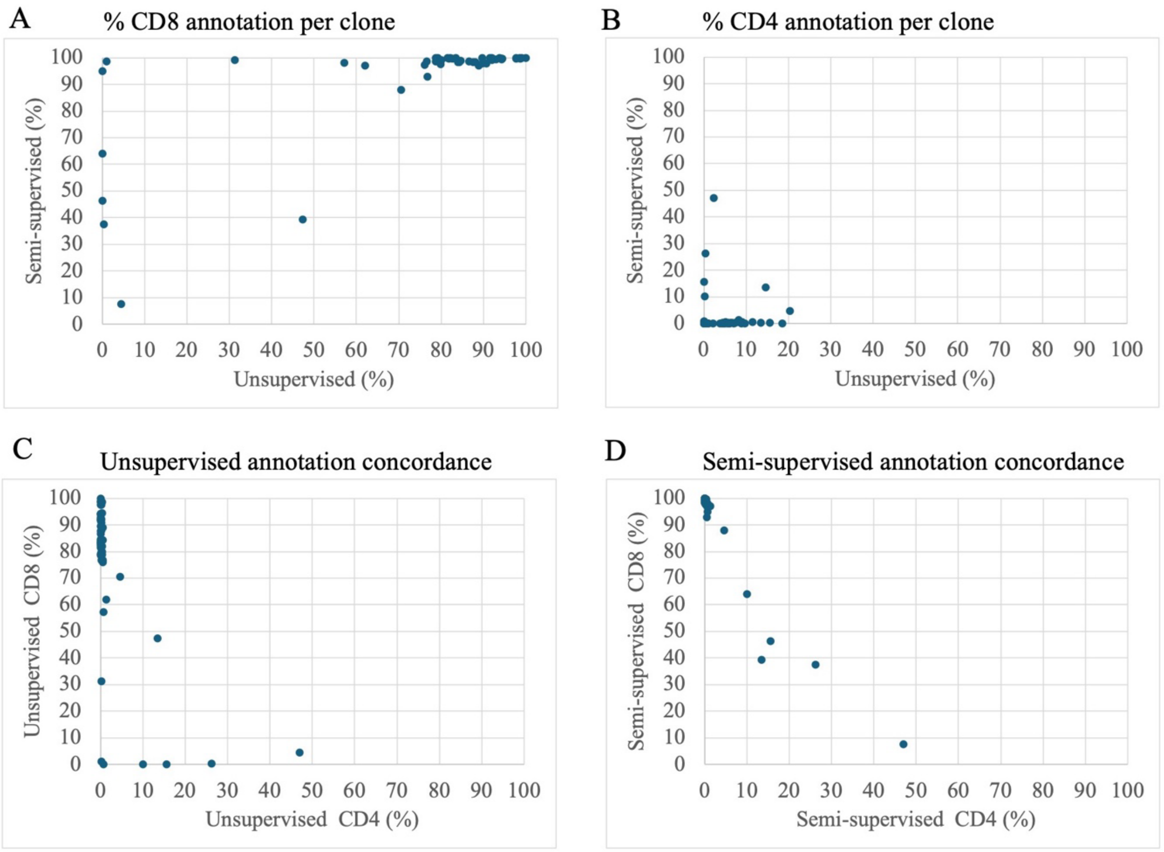

Supplementary Figure S7. Top 50 clones' annotation of the major T cell marker. (**A**) CD8 and (**B**) CD4**.** We also compared the CD8 and CD4 percentages for (**C**) unsupervised and (**D**) semi-supervised clustering. (**A-B**) The x-axis is the percentage of cells expressing for the cluster-based annotation. The y-axis represents the percentage of cells expressing the CD8 semi-supervised based annotation. (**C-D**) x-axis represents the % CD4 expression and y-axis is the % of CD8 expression. Each dot represents a unique clone.

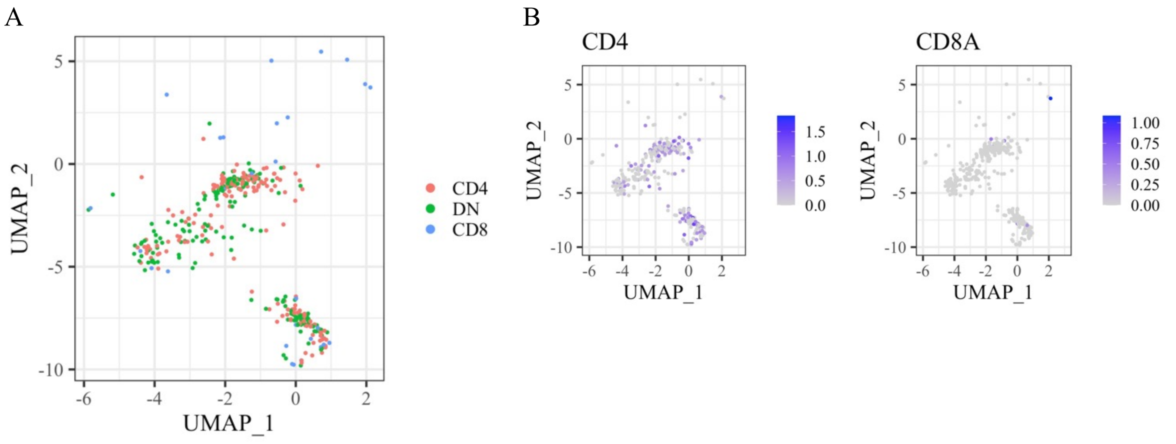

Supplementary Figure S8. Likely CD4+ expanded clone: TRAV13-1.TRAJ28 CAATDSGAGSYQLTF & TRBV20-1.TRBJ2-1 CSAPLGTSNEQFF. (**A**) The unsupervised clustered for this clone was identified in 11 different clusters. (**B**) Expression from left to right CD4 and CD8A.

R3 identification of MAIT cells with TCR-seq

MAIT cells traditionally have been identified based on the GEx alone annotation modelling that requires expression of TRAV1-2, KLRB1 and/or SLC4A10. To assess the enrichment of MAIT cells we first interrogated the standard GEx alone method using the unsupervised cluster-based annotation and FindMarker enrichment comparing the 25 clusters (resolution = 1). Only cluster 22 had significant enrichment of TRAV1-2 and KLRB1 expression and was specific to the Lewy body dementia (LBD) with 3.2% of their total cells (**Suppl. Fig. S9A**). Next, we extracted the scaled expression of the TRAV1-2 gene to showcase that every cluster had this variable gene (**Suppl. Fig. S9B**). Instead of using the GEx, we used the TCR-seq to identify the possible presence of MAIT cells using the TCR-seq based on the TRAV1-2 and J33/20/12. Interestingly, all data sets likely contained MAIT cell (GeoMean = 0.9%; SD±5.4%) with up to 19.3% of cells were likely MAIT cells in LBD (**Suppl. Fig. S9C**). Overall, using the TCR-seq was better able to identify the presence of the MAIT population.

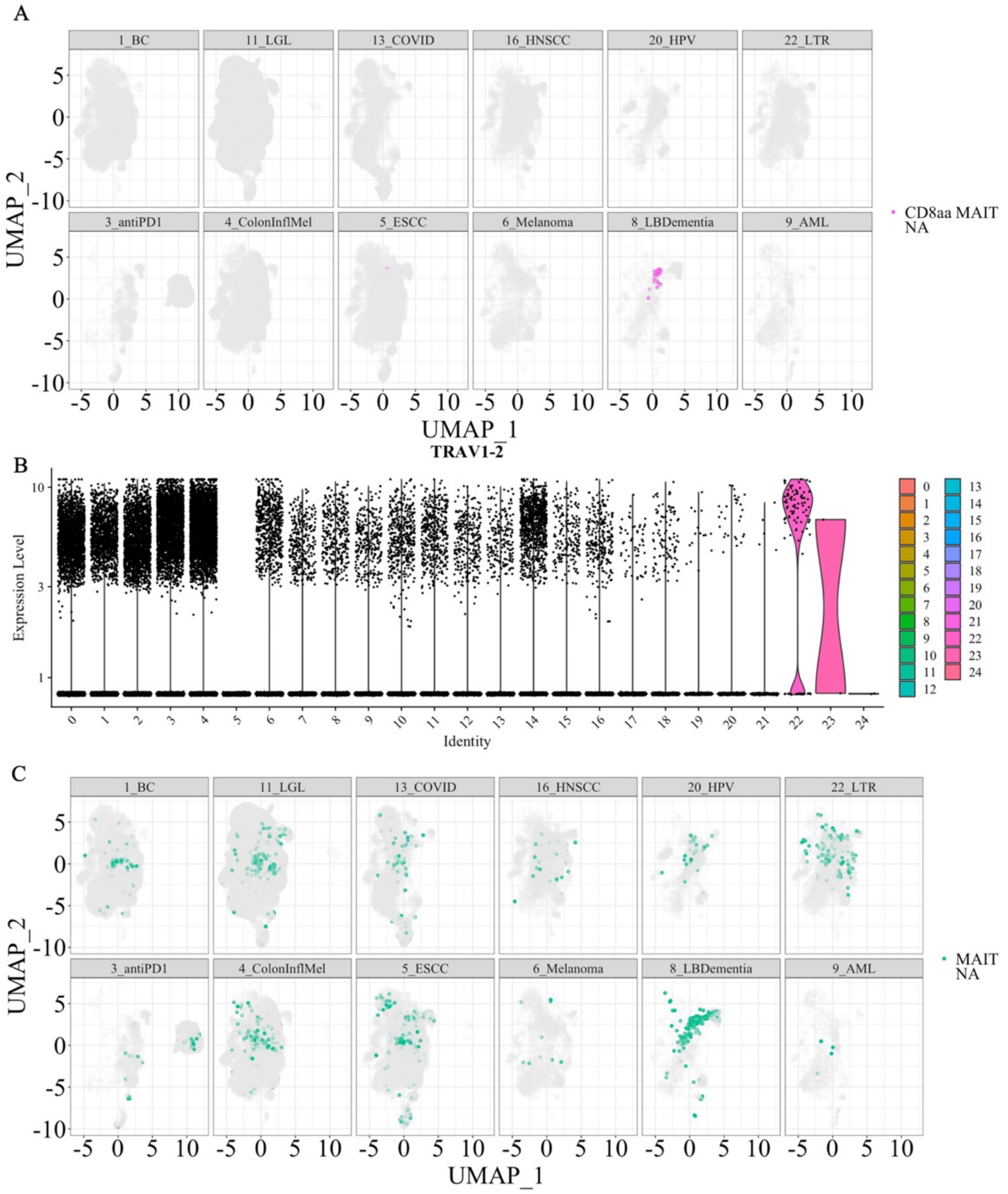

Supplementary Figure S9. Identification of MAIT cells. (**A**) UMAP plot of the Unsupervised clustering that was split by the different studies. This graph showcasing that only the Lewy body (LB) dementia had a cluster of MAIT cells identified based on TRAV1-2 and KLRB1 expression. (**B**) Scaled gene expression of TRAV1-2 from the 12 studies was present in each of the clusters. (**C**) TCRseq MAIT annotation based on TRAV1-2 TRAJ33/12/20 were identified in every study.

R4 Identifying epitope-specific TCR and/or biomarkers that warrant functional validation.
Unlike the above example that uses epitope prediction, this section explored the tetramer sorted dataset, as there is higher confidence that the TCR's will be epitope specific.

Some studies use tetramer sorting and various markers to identify clones and/or biomarkers of interest. The clone(s) of interest may not necessarily be the most expanded but are identified across different conditions with a distinct transcriptional profile. To illustrate this concept, we focused on a head and neck squamous cell carcinoma (HNSCC (*19*): GSE180268) dataset and used the remaining T cell atlas as the background to ensure signal enrichment when using the "FindMarker" statistic. In the original study, the authors state a clear narrative based on all single-cell and experimental data that PD-1 may be a potential target to boost the T cell immunity in HNSCC and concluded targeting PD-1 may be a good therapy to restore T cell function (*19*). The original analysis did not have an in-depth look at the TCR-seq to identify candidate TCRs that could have potential for screening, vaccine candidates and/or TCR-based therapies e.g., TCR-T.

Reanalysis of the T-cell receptor HNSCC data with STEGO.R revealed only a small number of public clones, which exhibited limited clonal expansion and lacked specificity towards disease (**Figure 4A; Suppl. Table S13**). Subsequently, we investigated the presence of clones shared across various treatments and conditions within individual patients and their clonal expansion. Specifically, as this dataset was tetramer sorted, we could deduce the TCR sequences likely able to recognize one of two HPV-epitopes HLA-A*01:01–HPV $E2_{329–337}$ (peptide sequence KSAIVTLTY;KSA), and HLA-A*01:01–HPV $E2_{151–159}$ (peptide sequence QVDYYGLYY; QVD)(*19*). There were clones that were KSA- and QVD-associated TCRs that were also identified in the PD-1 sort of primary tumor and metastatic lymph node (metLN) samples (**Suppl. Fig. S10A**). There was one KSA-specific clone in both HPV7 (**Suppl. Fig. S10B**) and HPV34 (**Suppl. Fig. S10C**). Additionally, HPV34 also had multiple QVD-specific clones (**Suppl. Fig. S10D; Suppl. Table S14**). Interestingly the QVD-specific HPV34 repertoire had many clones with TRAV21.TRAJ9 CAVDTGGFKTIF (n=11/27) and paired with different TCRβ arrangements.

We next compared if there were any significantly enriched genes common across the epitope-specific clones compared to the T-cell atlas for the above mentioned TCR's of interest. There were 243 genes enrichment common to those TCRs (two KSA and pooled QVD clones), including CXCL13, LINC02446, RACK1, TRIR, RBPJ, HLA-DQA2 and DUSP4 (**Suppl. Table S15**). This indicates a T cell signature with consistent transcriptional markers from the epitope-specific T-cells that were also present in the PD-1 sort.

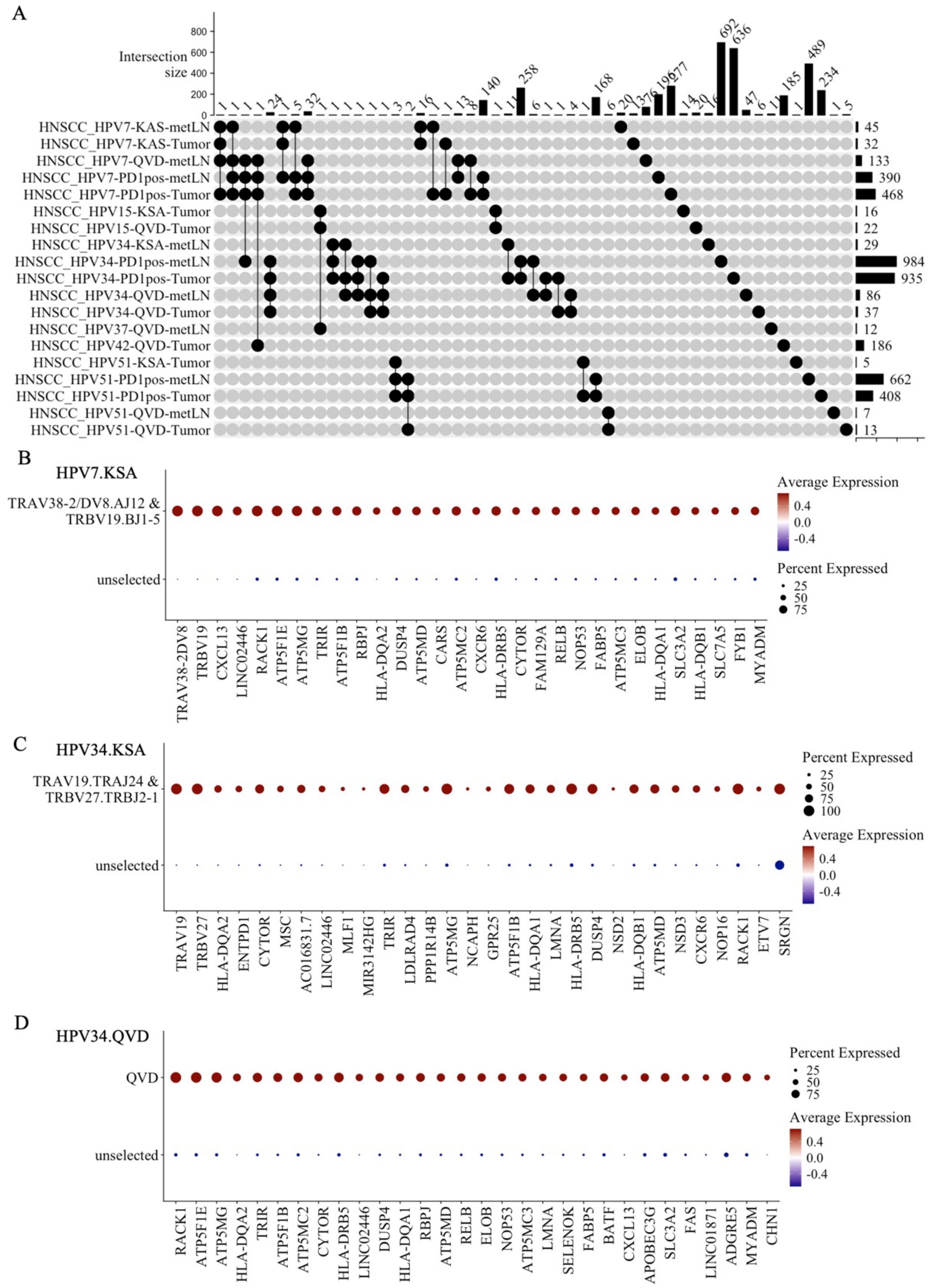

Supplementary Figure S10. Re-examining the HNSCC identifies novel transcriptional signature of the HPV-epitope and PD-1 specific clones from HPV34. (**A**) Upset plot showcasing the unique overlapping sequences i.e., paired αβTCR with the CDR3 sequence, representing HPV7, HPV15, HPV34 and HPV51 with various sorting conditions (KSA, QVD, PD-1) and tissues (Tumor or metastatic lymph node[metLN]). The dot represents if a sequence was present in that sample. The lines connect the overlapping samples. The top bar-plot represents the number of overlap the samples. The right bar plot is the total unique sequences per sample. (**B-D**) Dot plots representing the top 30 significant genes associated from most significance (left to right) for (**B**) HPV7-KSA clones (TRAV38-2/DV8.TRAJ12 CAYNCPEPSDSSYKLIF & TRBV19.TRBJ1-5 CASSMLLNQPQHF) that overlap with both PD-1 from metLN and Tumor, (**C**) HPV34-KSA (TRAV19.TRAJ24 CALSGTDSWGKLQF & TRBV27.TRBJ2-1 CASSLSGTLGNEQFF) clone overlapping with PD-1 from metLN and Tumor and (**D**) pooled QVD-specific clones from HPV34 that were also identified in the PD-1+ from metLN and Tumor sorts.

R5 Identified limited public clones and common clusters in the colon dataset

From the colon derived T cells, there were 38,394 unique clones across the 22 individuals in the dataset with most clones being private. Indeed, only 49 clones were shared among two or more individuals (**Suppl. Fig. S11**). Of these, 14 clonotypes were present in more than one disease state (**Suppl. Table S16**). Nine overlapped between the melanoma patients. Four overlapped in the normal controls and colitis, three of which were expanded in the colitis cases. Although the degree of expansion in these overlapping clones was minimal (n<10 per clone).

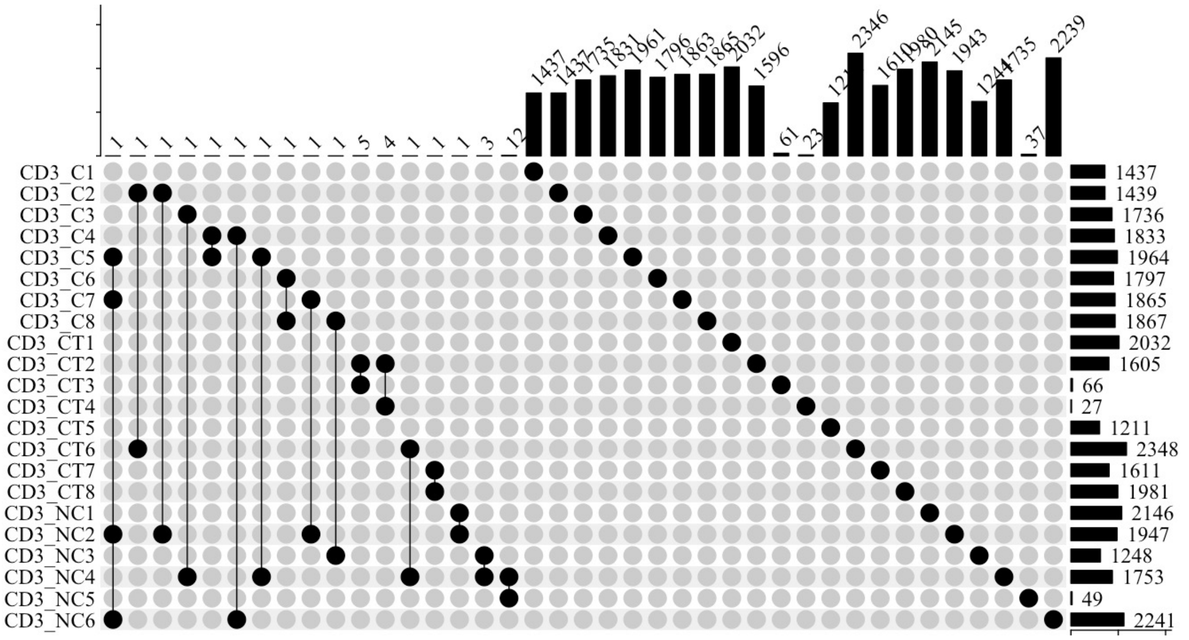

Supplementary Figure S11. Colitis datasets showcase unique TCR sequence overlap. The data is displayed as an UPSET plot. A dot indicates the presence of a unique clone. The line represents if there was any overlap across samples. (**top**) Bar graph represents the number of overlapping unique clone sequences that could be from multiple samples. (**right**) Bar graph representing the number of unique clones per sample. The uniqueness of the clones is based on the V(D)J arrangement of both chains and the unique CDR3 sequence of both chains. This plot includes both αβTCR and γδTCR lineages. C: colitis; NC: non-colitis; CT: normal controls.

We did not observe any clones that were specific to the cases experiencing colitis complication. Additionally, the repertoires of these individuals were also mostly private. Therefore, potential sequence similarity rather than exact matching may reveal TCR motifs more associated to a specific group (e.g., melanoma cases, no colitis etc.). To this end, TCR clustering with clusTCR2 was applied, resulting in the identification of 1607 alpha and 322 beta clusters (**Suppl. Table S4**), as well as 77 gamma and four delta clusters (**Suppl. Table S5**) that were present in one individual and a minimum of three clones.

Several clusters were found to be common among multiple individuals. These clusters included gamma clusters: TRGV9-TRGJP (n=21) and TRGV4-TRGJ2 (n=17) and alpha clusters TRAV1-2-TRAJ33 (n=18) and TRAV17-TRAJ54 (n=15). Despite their publicity, these patterns were not disease specific as they were observed across individuals with the different conditions. Indeed, the TRGV4-TRGJ2 is an expected cluster, as Vδ1γ4+ T cells are commonly observed in the colon (*63*). Similarly, TRGV9-TRGJP, commonly associated with the invariant Vδ2γ9+ T cells, is known to regularly interact with (E)-4-hydroxy-3-methyl-but-2-enyl pyrophosphate (HMBPP), a common microbial phosphoantigen (*64*). The common alpha and beta clusters will be discussed in the context of the global analysis in **the main results section**.

In conclusion, the identification of these gamma clusters is not unexpected in gut-derived T cells.

R6 Identification of disease specific phenotypes with semi-supervised annotation method

There is risk of not finding T-cell disease-specific patterns when interrogating a small number of cells or limited expression diversity due to experimental protocols (e.g., epitope-specific, tetramer sorting etc.). This is because T-cell functions are defined in contrast to other T-cell phenotypes, due to the minute variations that guide these differences. Consequently, identifying the specific signature may be missed due to expressional similarity or sparsity of the overall data present. This in turn limits our capacity to find the disease-specific/epitope-specific signature for any given TCR sequence. To overcome this challenge, we identified that combining all 12 datasets with 143 unique samples from 90 individuals allowed for identification of 49 distinct T cell phenotypes, including Th2 cells and DN populations that have been previously under-represented in studies. We examined the proportion of total CD8, CD4 and double negative (DN) T cells present. From the protein experience, specifically PBMCs, we would expect a bias of CD8>CD4> DN. Yet, this may not be the expected pattern when examining the transcriptome. The higher prevalence of DN is likely due to the poor coverage of the CD4+ marker. So researchers need to be cautions that the DN may be CD4+ T cells (**Suppl. Table S17**).

By combining the data, we could also interrogate stratification of the T cell population. The most common cell types (>5%) identified included (mean±standard error of the mean[SEM]; range): CD8ab+ Eff (25.0±2.6%; 12.3-38.5%), CD8ab+ Eff Tc1 (12.4±2.0%; 1 2.9-25.2%), CD8ab+ Eff Tc9 (5.9±2.0%; 0.4-24.0%), DN Naïve (7.0±1.2%; 0.4-14.5%) and DN FTH1 (5.5±1.2%; 0.4-14.2%).

There was substantial variation of the T cell sub-populations across the datasets. As expected, the tetramer (HPV epitope) sorted HNSCC was mostly CD8+ T cells (~97.0%) with less than 1% CD4+ T cells. Intriguingly, total naïve cell population were prevalent in breast cancer, COVID (lung tissue), T cell cancer (LGL), cervical cancer, Lewy body dementia (LBD) and acute myeloid leukemia (AML) (range 9.9% to 15.2%). Lastly there were considerable proportions of Tregs of both CD4+ and DN. The DN's are likely to be CD4+ T cells, as the CD4 marker was poorly captured in 10x Genomics datasets. Tregs were highly prevalent (>8%) in breast cancer, esophageal cancer and melanoma, moderately present (5% to 8%) in colitis, LBD, anti-PD1 treated cancers, and lower percentage (<3%) in lung transplantation, HPV, COVID-19 lung tissue, LGL and AML.

R7 Public clones are likely related to common infections.

Given that we had access to 12 T-cell focused datasets, we next aimed to identify if there were any public clones across those studies, and if they could be present due to common histories (e.g., common viral infections). 53 of the ~250,000 unique clones were present in two or more studies (**Suppl. Table S18**). To further understand if these clones were common because of shared history (e.g., viral/bacterial exposure), we annotated these 53 αβTCRs with the DETECT tool for their epitope-specificity. 13 clones with a threshold of 0.2 (~0.99 probability) with high confidence of epitope specificity (**Suppl. Table S19**). This process identified nine TCRs with specificity for Human gammaherpesvirus 4 (Epstein–Barr virus [EBV]) for CLGGLLTMV, FLYALALLL, GLCTLVAML and YLRGRAYGL, three for Human betaherpesvirus 5 (Cytomegalovirus [CMV]) for NLVPMVATV and RPHERNGFTVL and one for Influenza A virus GILGLVFTL. The IMW DETECT could also identify eight clones

that were likely MAIT cells as they were predicted to interact with MR1:5-OP-RU. The remaining 40 clones had unknown specificity.

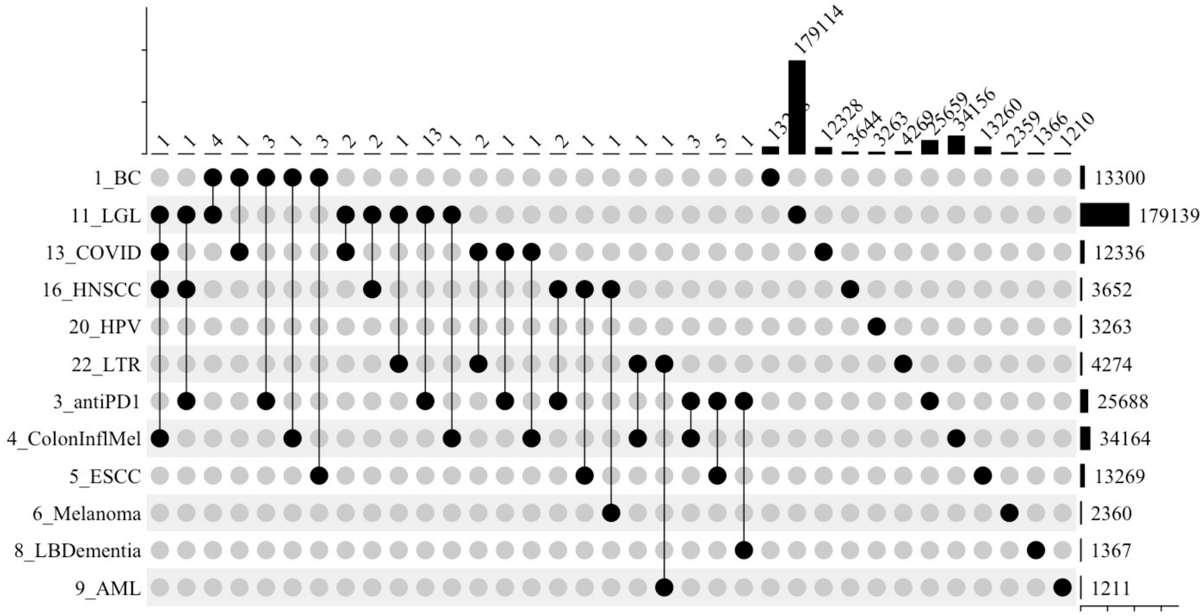

Supplementary Figure S12. Overlap of clones across the 12 datasets. (**top**) Bar graph represents the number of overlapping unique clone sequences that could be from multiple studies. (**right**) Bar graph representing the number of unique clones per study. The uniqueness of the clones is based on the V(D)J arrangement of both chains and the unique CDR3 sequence of both chains. This plot includes the αβTCR as the γδTCR were only present in the 4_ColonInflamMel data set.

Section S3

This supplementary discussion covers additional points related to single cell GEx analysis limitations as well as some dataset-specific insights.

In the single cell field, there is an assumption that unsupervised clustering captures the underlying cellular architecture (65, 66). However, while this works for cellular populations with a unidirectional fate, T cells display a wide variation in distinct subtypes and states. This is best illustrated by the diffusion of the major T cell markers, CD4 and CD8A, across all clusters whereas these should form two distinct populations. Interrogation of the top 50 most expanded clones showed that the semi-supervised annotations outperformed the unsupervised clustering as there were fewer cells' mis-identified given that CD4 expression excludes CD8 during thymic development. Additionally, as T cells function is directly related to their unique TCR, using the paired TCR also allowed accurate delineation of the γδ T cells , which can be CD8+ (up to 30%) (67), as well as the invariant T cells, such as the MAIT cell population. Therefore, the most accurate T-cell classification based on the RNA level could be accomplished by a combination of semi-supervised annotations with TCR-seq.

One of the common analysis strategies used to interrogate single cell data involves pseudotime analysis of cell states (68). Based on the Monocle 3 package, defining the pseudotime from an immature cell to a terminally differentiated cell requires utilizing the clustering and UMAP (21). This has shown some utility in developmental setting where the undifferentiated cells and differentiated cells transcriptional signatures segregate out (69). However, T-cells functions are highly dynamic with a bidirectional fluidity between phenotypes. In addition, as discussed in the previous paragraph, unsupervised clustering fails at identifying T-cell subtypes and states. Therefore, the main assumption for pseudotime analysis cannot be met. An alternative strategy better suited towards T-cells involves the interrogation of the expanded TCR vs singlets (unique TCRs only), where the latter are likely to not be involved in the pathology.

Many of the studies from which the T-cell atlas was derived lacked detail of what markers were used to identify each T-cell subpopulation (18-20, 23, 26, 28-36). So, to improve the reproducibility and consistency in the field, we proposed robust annotation strategies with the semi-automated gating-like annotation strategy with scGate (42), and integrated them in the STEGO.R tool. This process relies on defining a fixed marker set (supervised aspect of the modelling), and levels (gating order) to find the population of interest. This is then coupled in a next step with any information provided by the TCR. To our knowledge, this is the first strategy that uses a joint GEx and TCR approach for annotation purposes. For future use, we uploaded the annotated 500K TCR-GEx.rds object to Zenodo as a T cell atlas, so that it can be used to overlay and transfer the annotations to a new data set.

We used the GEx semi-supervised annotation and identified some qualitative changes in the proportions of the different T cell populations. One notable finding was the changing prevalence of Tregs. Here we identified that many of the cancer samples had moderate to high levels of Tregs including breast cancer, esophageal cancer, head and neck cancer and melanoma as well as various cancer samples under anti-PD1 treatment. Tregs are a T cell subset that induce self-tolerance, but can in cancer settings suppress anti-tumor immunity (70). This is because Tregs that have IL-10 expression influence exhaustion in their CD4 and CD8+ T cell counterparts (71). An advanced gastric cancer that used anti-PD-1 observed that ~10% of cases have hyperprogressive disease, and was associated with an increasing the Tregs

population ([72]). Given the influence of Tregs in some of the more metastatic cancer, there may be a benefit to target the Treg population or its cytokine that signals exhaustion i.e., target IL-10. Overall, being able to compare many datasets enabled identification of disease-specific fluctuations in the T cell populations.

A broader issue in single cell studies is finding the enrichment signal, especially in smaller or focused studies, where the cells of interest are too similar to the background data. To demonstrate the loss of disease specificity, we re-analyzed the lung transplantation recipient (LTR) dataset. The overall profile of the top expanded clones compared to the remainder of the data set showed that these clones were cytotoxic (GZMB) and in the late activation stage (HLA-DR). However, when this clone was compared to the T cell Atlas, a distinct pattern emerged with several detoxification genes including the Metallothioneins MT1E, MT1F and MT1X. These markers have been well studied in regulatory T cells where high MT1 expression has been linked to lower IL-10 expression ([22]). A recent single cell study identified that these genes' expression in CD8+ T cells were linked to anti-PD1 treatment resistance in esophageal cancer ([73]). These Metallothioneins are therefore strong leads for future follow-up studies, which were missed in the original LTR study as the expression level within the captured cell population was too similar. This showcases the advantage of using an integrated T cell Atlas for identifying potential biomarkers and differential signals.

Tetramer-sorted samples have the benefit of knowing which HLA and epitope are likely interacting with the pool of T cells. To showcase a process of prioritizing T cells and their associated biomarkers for functional validation, we re-interrogated the HNSCC data set that had been PD-1 and tetramer sorted for two HLA-A*01:01+ HPV-epitopes([19]). Our approach identified pools of PD-1+ HPV-specific T cells and identified several common transcripts including RACK1 and LINC02446. RACK1 has been proposed as a regulator of T cell homeostasis ([74]) and may be involved in activating CD4+ T cells ([75]). While little is known about the LINC02446 transcript, recent evidence implicates that it may be a promising therapeutic target in bladder cancer ([76]). In addition, we managed to prioritize a list of 27 unique TCR sequences, which is a feasible list for next stage functional testing.

The main focus of most T cell studies has been the conventional αβTCR, with fewer studies focusing on gamma-delta (γδ) TCRs. In recent decades, γδ T cells are emerging as a critical T cell lineage to interrogate as they have diverse innate- and adaptive-like functions([77]), have a higher prevalence in mucosal membranes, and are emerging as the basis for novel therapies in cancer (reviewed in ([78])). The difficulties of researching γδ T cells populations are their culturing conditions, their lower frequency in PBMCs, and their mostly unknown non-peptide-based epitopes. Yet, these barriers to interrogate γδTCR do not exist when using single cell transcriptomics. Nonetheless, γδ-T cells are rarely interrogated in single cell studies as they are not included in the standard V(D)J kit (10x Genomics), and their processing is often poor optimized in the downstream alignment processes (e.g. cell Ranger vdj alignment). While methods like TRUST4 may be used to partially recover γδ clones from scRNA-seq data, even without the inclusion of γδTCR primers, coverage remains limited at best([79]). When assembling the current T cell Atlas starting from ~1.13M T cells, only ~500K could be paired with a functional TCR. When looking at the independent studies, we could see that the 10x Genomics efficiency of finding cells that had both GEx and TCR data was on average less than 50%. Part of the missingness in the data is likely due not sequencing γδTCRs, which are a considerable fraction of the T-cell repertoire independent of the sample.

There is a major advantage to sequencing all four TCR chains to capture both lineages, especially when interrogating tissues where γδ T cells are more abundant. An example can be found in the colitis complication data set, which included all four chains, as the gut is known to be enriched for γδTCR populations (26). Our analysis identified melanoma-specific TRGV4+ γδ T cells that expressed a CD8ab+ T cell profile. There is limited, but emerging evidence that TRGV4+ γδ T cells that express the CD8ab+ heterodimer can target tumor epitopes presented on common HLA-A molecules(44, 45). It is therefore possible that the γδ TCR's identified in the melanoma-cases in gut could be melanoma-peptide restricted. These findings further call into question the importance of separating these lineages (αβTCR vs γδTCR) from a functional context, given that both lineages can have epitope-specificity to peptides, lipids and small molecules.

While TCR sequence are the result of a random recombination event and are mostly unique to the individual from which they are derived, it is known that there are common public TCR clonotypes across individuals. Using our T cell Atlas, we were able to identify a set of 53 TCR sequences that were present in two or more studies. A third of these common TCR sequences could be annotated as being EBV, CMV or MAIT related. To further understand the presence of global patterns across TCR repertoires, we interrogated the TCR sequence similarity across clones and samples that was unlikely the result of chance or generation probability. In addition to a TRAV1-2 TRAJ33 cluster, which were likely MAIT cells, we also identified two common clusters, denoted by their use of TRAV3 and TRAV26-1. Further investigation revealed that the TRAV3 cluster in particular likely represents a shared disease history, which is currently still unknown.

Legends for tables S1 to S19.

The "**Supplementary_tables.xlsx**" contains the 19 supplementary tables for both the main and supplementary material.

Supplementary Table S1. Publicly available datasets to test STEGO (10x Genomics based data)
Supplementary Table S2. Clones either more prevalent in treatment or acute cellular rejection (ACR)
Supplementary Table S3. TCR's from the T-cell Atlas predicted to be epitope specific with IMW-DETECT
Supplementary Table S4. The colitis dataset TRA and TRB clusters
Supplementary Table S5. The colitis dataset TRG and TRD clusters
Supplementary Table S6. Results of the TCR neighbor enrichment analysis using clustcrdist for the colitis dataset
Supplementary Table S7. Results of the TCR neighbor enrichment analysis using clustcrdist for TRBV6-2 clones from C3 clusters
Supplementary Table S8. Epitope prediction of the alpha clusters using IMW-DETECT
Supplementary Table S9. Results of the TCR neighbor enrichment analysis using clustcrdist (T-cell Atlas)
Supplementary Table S10. T cell functions annotation profile within STEGO.R application
Supplementary Table S11. Percentage of the annotations for TRAV12-2.TRAJ9_CAATTGGFKTIF & TRBV20-1.TRBD2.TRBJ2-4_CSATEGNIQYF

Supplementary Table S12. Comparing the unsupervised to semi-supervised annotations for the major T cell markers CD4 and CD8
Supplementary Table S13. Head and neck cancer TCR-seq summary table
Supplementary Table S14. List of clones overlapping with both PD-1 and QVD in HPV34
Supplementary Table S15. Significant genes overlapped with KSA- and QVD-specific TCR from HPV7 and HPV34
Supplementary Table S16. The 14 clones that overlapped two diseases in the colitis dataset
Supplementary Table S17. Average annotation for each of the 12 datasets
Supplementary Table S18. Overlapping TCR across disease types
Supplementary Table S19. Epitope prediction of the 39 clones using IMW-DETECT

Supplementary Table S20. Input data required for 10x Genomics and BD Rhapsody.

Source	Inputs
10x Genomics	Features.csv.gz: Gene ID (SYMBOL)<br>Barcode.csv.gz: Cell ID (AGGATTT_1)<br>Matrix.mtx.gz: Gene expression.<br>Contig file (AIRR) format: contains the V(D)J sequences
BD Rhapsody	Features.csv.gz: Gene ID (SYMBOL)<br>Barcode.csv.gz: Cell ID (numeric)<br>Matrix.mtx.gz: expression captured<br>Sample_Tag.csv: multiplexing annotations<br>Unfiltered contig file (AIRR) format or Dominant (AIRR): contains the V(D)J sequencest

Supplementary Table S21. Structure of director and purpose of each folder

**File**	**Inputs**
0_rawfiles	Copy the raw 10x Genomics/BD Rhapsody outputs.
1_ClusTCR2	Storage of the alpha-gamma (AG) or beta-delta (BD) clusters for each sample.<br>Store the merged AG_ or BD_ file for the clustering.
1_SeuratObj	Contains the matrix file (cell x gene) and the meta data that contains the TCR information.
1_TCR_Explore	Contains the (.csv) TCR_Explore files which can be used in the webapp https://tcr-explore.erc.monash.edu for visualizing the repertoire alone.
1_TCRex	Contains the (.tsv) file that are in the required format for predicting epitope specificity in the TCRex webtool.
2_ClusTCR2	Merged clusTCR2 files
2_TCRex	Merged TCRex files
3_SCobj	a. Filtered Seurat objects<br>b. Merged and batch corrected<br>c. Annotated file<br>d. Removal of unwanted samples (also copy to 4_analysis file).<br>e. Reformatting from scRepertoire

File	Inputs
4_Analysis	This file stores all the outputs required for the STEGO.R analysis section.<br>This includes annotated_seurat.rds, AG_cluster.csv, BD_cluster.csv, TCRex.tsv, and IMW-DETECT.tsv file<br>The folder also include .csv file for updating the ID's as required.
custom_db	Contains nine folders that can be used by scGate for creating new annotation strategies. 'master_table.tsv' contains the 'name' and 'signature' required for all the annotation strategies. The specific strategies are in the files with the suffix '_scGate_model.tsv'. This suffix is required to identify each annotation label. Only use numbers and letters with no spaces for the file name.
Figures.Tables	Save the manual output files if desired
Prioritization	This directory will contain the automated outputs of the analysis for a first pass look over the results.<br>Contains the following sub-directories:<br>Clustering<br>• A<br>• G<br>• B<br>• D<br>EpitopePred<br>• Can also be used for identifying TCR per annotation model<br>Multi<br>• PublicLike (for clones present in multiple samples)<br>• Unique (clones present in one sample only)<br>ImmunoDom (single sample)<br>PolyClonal (single sample)

REFERENCES AND NOTES

1. S. Valkiers, N. de Vrij, S. Gielis, S. Verbandt, B. Ogunjimi, K. Laukens, P. Meysman, Recent advances in T-cell receptor repertoire analysis: Bridging the gap with multimodal single-cell RNA sequencing. *ImmunoInformatics* **5**, 100009 (2022).

2. S. Valkiers, M. Van Houcke, K. Laukens, P. Meysman, ClusTCR: A python interface for rapid clustering of large sets of CDR3 sequences with unknown antigen specificity. *Bioinformatics* **37**, 4865–4867 (2021).

3. K. Mayer-Blackwell, S. Schattgen, L. Cohen-Lavi, J. C. Crawford, A. Souquette, J. A. Gaevert, T. Hertz, P. G. Thomas, P. Bradley, A. Fiore-Gartland, TCR meta-clonotypes for biomarker discovery with *tcrdist3* enabled identification of public, HLA-restricted clusters of SARS-CoV-2 TCRs. *eLife* **10**, e68605 (2021).

4. D. Hudson, A. Lubbock, M. Basham, H. Koohy, A comparison of clustering models for inference of T cell receptor antigen specificity. *ImmunoInformatics* **13**, 100033 (2024).

5. J. Glanville, H. Huang, A. Nau, O. Hatton, L. E. Wagar, F. Rubelt, X. Ji, A. Han, S. M. Krams, C. Pettus, N. Haas, C. S. L. Arlehamn, A. Sette, S. D. Boyd, T. J. Scriba, O. M. Martinez, M. M. Davis, Identifying specificity groups in the T cell receptor repertoire. *Nature* **547**, 94–98 (2017).

6. S. Gielis, P. Moris, W. Bittremieux, N. De Neuter, B. Ogunjimi, K. Laukens, P. Meysman, Detection of enriched T cell epitope specificity in full t cell receptor sequence repertoires. *Front. Immunol.* **10**, 2820 (2019).

7. A. Montemurro, V. Schuster, H. R. Povlsen, A. K. Bentzen, V. Jurtz, W. D. Chronister, A. Crinklaw, S. R. Hadrup, O. Winther, B. Peters, L. E. Jessen, M. Nielsen, NetTCR-2.0 enables accurate prediction of TCR-peptide binding by using paired TCRα and β sequence data. *Commun. Biol.* **4**, 1060 (2021).

8. G. Croce, S. Bobisse, D. L. Moreno, J. Schmidt, P. Guillame, A. Harari, D. Gfeller, Deep learning predictions of TCR-epitope interactions reveal epitope-specific chains in dual alpha T cells. *Nat. Commun.* **15**, 3211 (2024).

9. E. Rosati, C. M. Dowds, E. Liaskou, E. K. K. Henriksen, T. H. Karlsen, A. Franke, Overview of methodologies for T-cell receptor repertoire analysis. *BMC Biotechnol.* **17**, 61 (2017).

10. S. A. Schattgen, K. Guion, J. C. Crawford, A. Souquette, A. M. Barrio, M. J. T. Stubbington, P. G. Thomas, P. Bradley, Integrating T cell receptor sequences and transcriptional profiles by clonotype neighbor graph analysis (CoNGA). *Nat. Biotechnol.* **40**, 54–63 (2022).

11. F. Drost, Y. An, L. M. Dratva, R. G. H. Lindeboom, M. Haniffa, S. A. Teichmann, F. Theis, M. Lotfollahi, B. Schubert, Integrating T-cell receptor and transcriptome for 4 large-scale single-cell immune profiling analysis. bioRxiv 449733 [Preprint] (2022).

12. S. Becattini, D. Latorre, F. Mele, M. Foglierini, C. De Gregorio, A. Cassotta, B. Fernandez, S. Kelderman, T. N. Schumacher, D. Corti, A. Lanzavecchia, F. Sallusto, T cell immunity. Functional heterogeneity of human memory $CD4^+$ T cell clones primed by pathogens or vaccines. *Science* **347**, 400–406 (2015).

13. Y. Hao, T. Stuart, M. H. Kowalski, S. Choudhary, P. Hoffman, A. Hartman, A. Srivastava, G. Molla, S. Madad, C. Fernandez-Granda, R. Satija, Dictionary learning for integrative, multimodal and scalable single-cell analysis. *Nat. Biotechnol.* **42**, 293–304 (2023).

14. F. A. Wolf, P. Angerer, F. J. Theis, SCANPY: Large-scale single-cell gene expression data analysis. *Genome Biol.* **19**, 15 (2018).

15. K. Verstaen, K. Ibech, I. Lammens, J. Roels, Y. Saeys, B. N. Lambrecht, N. Vandamme, S. Vanhee, DALI (Diversity AnaLysis Interface): a novel tool for the integrated analysis of multimodal DALI (Diversity AnaLysis Interface): A novel tool for the integrated analysis of multimodal. bioRxiv 471549 [Preprint] (2022).

16. N. Borcherding, N. L. Bormann, G. Kraus, Kraus, scRepertoire: An R-based toolkit for single-cell immune receptor analysis. *F1000Res.* **9**, 47 (2020).

17. K. A. Mullan, N. de Vrij, S. Valkiers, P. Meysman, Current annotation strategies for T cell phenotyping of single-cell RNA-seq data. *Front. Immunol.* **14**, 1306169 (2023).

18. M. E. Snyder, K. Moghbeli, A. Bondonese, A. Craig, I. Popescu, L. Fan, T. Tabib, R. Lafyatis, K. Chen, H. E. Trejo Bittar, E. Lendermon, J. Pilewski, B. Johnson, S. Kilaru, Y. Zhang, P. G. Sanchez, J. K. Alder, P. A. Sims, J. F. McDyer, Modulation of tissue resident memory T cells by glucocorticoids after acute cellular rejection in lung transplantation. *J. Exp. Med.* **219**, e20212059 (2022).

19. C. S. Eberhardt, H. T. Kissick, M. R. Patel, M. A. Cardenas, N. Prokhnevska, R. C. Obeng, T. H. Nasti, C. C. Griffith, S. J. Im, X. Wang, D. M. Shin, M. Carrington, Z. G. Chen, J. Sidney, A. Sette, N. F. Saba, A. Wieland, R. Ahmed, Functional HPV-specific PD-1$^+$ stem-like CD8 T cells in head and neck cancer. *Nature* **597**, 279–284 (2021).

20. Y. Zheng, Z. Chen, Y. Han, L. Han, X. Zou, B. Zhou, R. Hu, J. Hao, S. Bai, H. Xiao, W. V. Li, A. Bueker, Y. Ma, G. Xie, J. Yang, S. Chen, H. Li, J. Cao, L. Shen, Immune suppressive landscape in the human esophageal squamous cell carcinoma microenvironment. *Nat. Commun.* **11**, 6268 (2020).

21. C. Trapnell, D. Cacchiarelli, J. Grimsby, P. Pokharel, S. Li, M. Morse, N. J. Lennon, K. J. Livak, T. S. Mikkelsen, J. L. Rinn, The dynamics and regulators of cell fate decisions are revealed by pseudotemporal ordering of single cells. *Nat. Biotechnol.* **32**, 381–386 (2014).

22. H. Dai, L. Wang, L. Li, Z. Huang, L. Ye, Metallothionein 1: A new spotlight on inflammatory diseases. *Front. Immunol.* **12**, 739918 (2021).

23. S. Gao, Z. Wu, B. Arnold, C. Diamond, S. Batchu, V. Giudice, L. Alemu, D. Q. Raffo, X. Feng, S. Kajigaya, J. Barrett, S. Ito, N. S. Young, Single-cell RNA sequencing coupled to TCR profiling of large granular lymphocyte leukemia T cells. *Nat. Commun.* **13**, 1982 (2022).

24. J. Shi, J. Zhou, X. Zhang, W. Hu, J. F. Zhao, S. Wang, F. S. Wang, J. Y. Zhang, Single-cell transcriptomic profiling of MAIT cells in patients with COVID-19. *Front. Immunol.* **12**, 700152 (2021).

25. P. Meysman, N. De Neuter, S. Gielis, D. Bui Thi, B. Ogunjimi, K. Laukens, On the viability of unsupervised T-cell receptor sequence clustering for epitope preference. *Bioinformatics* **35**, 1461–1468 (2019).

26. A. M. Luoma, S. Suo, H. L. Williams, T. Sharova, K. Sullivan, M. Manos, P. Bowling, F. S. Hodi, O. Rahma, R. J. Sullivan, G. M. Boland, J. A. Nowak, S. K. Dougan, M. Dougan, G.-C. Yuan, K. W. Wucherpfennig, Molecular pathways of colon inflammation induced by cancer immunotherapy. *Cell* **182**, 655–671.e22 (2020).

27. T. Guo, M. Y. Koo, Y. Kagoya, M. Anczurowski, C.-H. Wang, K. Saso, M. O. Butler, N. Hirano, A subset of human autoreactive CD1c-restricted T cells preferentially expresses TRBV4-1^+ TCRs. *J. Immunol.* **200**, 500–511 (2018).

28. E. Azizi, A. J. Carr, G. Plitas, A. E. Cornish, C. Konopacki, S. Prabhakaran, J. Nainys, K. Wu, V. Kiseliovas, M. Setty, K. Choi, R. M. Fromme, P. Dao, P. T. McKenney, R. C. Wasti, K. Kadaveru, L. Mazutis, A. Y. Rudensky, D. Pe'er, Single-cell map of diverse immune phenotypes in the breast tumor microenvironment. *Cell* **174**, 1293–1308.e36 (2018).

29. T. D. Wu, S. Madireddi, P. E. de Almeida, R. Banchereau, Y.-J. J. Chen, A. S. Chitre, E. Y. Chiang, H. Iftikhar, W. E. O'Gorman, A. Au-Yeung, C. Takahashi, L. D. Goldstein, C. Poon, S. Keerthivasan, D. E. de Almeida Nagata, X. Du, H.-M. Lee, K. L. Banta, S. Mariathasan, M. Das Thakur, M. A. Huseni, M. Ballinger, I. Estay, P. Caplazi, Z. Modrusan, L. Delamarre, I. Mellman, R. Bourgon, J. L. Grogan, Peripheral T cell expansion predicts tumour infiltration and clinical response. *Nature* **579**, 274–278 (2020).

30. K. L. Banta, X. Xu, A. S. Chitre, A. Au-Yeung, C. Takahashi, W. E. O'Gorman, T. D. Wu, S. Mittman, R. Cubas, L. Comps-Agrar, A. Fulzele, E. J. Bennett, J. L. Grogan, E. Hui, E. Y. Chiang, I. Mellman, Mechanistic convergence of the TIGIT and PD-1 inhibitory pathways necessitates co-blockade to optimize anti-tumor $CD8^+$ T cell responses. *Immunity* **55**, 512–526.e9 (2022).

31. L. Han, S. Chen, Z. Chen, B. Zhou, Y. Zheng, L. Shen, Interleukin 32 promotes Foxp3$^+$ Treg cell development and CD8$^+$ T cell function in human esophageal squamous cell carcinoma microenvironment. *Front. Cell Dev. Biol.* **9**, 704853 (2021).

32. K. M. Mahuron, J. M. Moreau, J. E. Glasgow, D. P. Boda, M. L. Pauli, V. Gouirand, L. Panjabi, R. Grewal, J. M. Luber, A. N. Mathur, R. M. Feldman, E. Shifrut, P. Mehta, M. M. Lowe, M. D. Alvarado, A. Marson, M. Singer, J. Wells, R. Jupp, A. I. Daud, M. D. Rosenblum, Layilin augments integrin activation to promote antitumor immunity. *J. Exp. Med.* **217**, e20192080 (2020).

33. T. Shibata, S. Shah, T. Evans, H. Coleman, B. J. Lieblong, H. J. Spencer, C. M. Quick, T. Sasagawa, O. W. Stephens, E. Peterson, D. Johann, Jr., Y.-C. Lu, M. Nakagawa, Expansion of human papillomavirus-specific T cells in periphery and cervix in a therapeutic vaccine recipient whose cervical high-grade squamous intraepithelial lesion regressed. *Front. Immunol.* **12**, 645299 (2021).

34. D. Gate, E. Tapp, O. Leventhal, M. Shahid, T. J. Nonninger, A. C. Yang, K. Strempfl, M. S. Unger, T. Fehlmann, H. Oh, D. Channappa, V. W. Henderson, A. Keller, L. Aigner, D. R. Galasko, M. M. Davis, K. L. Poston, T. Wyss-Coray, CD4$^+$ T cells contribute to neurodegeneration in Lewy body dementia. *Science* **374**, 868–874 (2021).

35. I. S. Cheon, C. Li, Y. M. Son, N. P. Goplen, Y. Wu, T. Cassmann, Z. Wang, X. Wei, J. Tang, Y. Li, H. Marlow, S. Hughes, L. Hammel, T. M. Cox, E. Goddery, K. Ayasoufi, D. Weiskopf, J. Boonyaratanakornkit, H. Dong, H. Li, R. Chakraborty, A. J. Johnson, E. Edell, J. J. Taylor, M. H. Kaplan, A. Sette, B. J. Bartholmai, R. Kern, R. Vassallo, J. Sun, Immune signatures underlying post-acute COVID-19 lung sequelae. *Sci. Immunol.* **6**, eabk1741 (2021).

36. L. Penter, S. H. Gohil, T. Huang, E. M. Thrash, D. Schmidt, S. Li, M. Severgnini, D. Neuberg, F. S. Hodi, K. J. Livak, R. Zeiser, P. Bachireddy, C. J. Wu, Coevolving JAK2^{V617F+}relapsed AML and donor T cells with PD-1 blockade after stem cell transplantation: an index case. *Blood Adv.* **5**, 4701–4709 (2021).

37. E. O. Karakaslar, N. Katiyar, M. Hasham, A. Youn, S. Sharma, C.-H. Chung, R. Marches, R. Korstanje, J. Banchereau, D. Ucar, Transcriptional activation of Jun and Fos members of the AP-1 complex is a conserved signature of immune aging that contributes to inflammaging. *Aging Cell* **22**, e13792 (2023).

38. Z. Sethna, Y. Elhanati, C. G. Callan, A. M. Walczak, T. Mora, OLGA: Fast computation of generation probabilities of B- and T-cell receptor amino acid sequences and motifs. *Bioinformatics* **35**, 2974–2981 (2019).

39. J. Neefjes, M. L. Jongsma, P. Paul, O. Bakke, Towards a systems understanding of MHC class I and MHC class II antigen presentation. *Nat. Rev. Immunol.* **11**, 823–836 (2011).

40. J. Cao, M. Spielmann, X. Qiu, X. Huang, D. M. Ibrahim, A. J. Hill, F. Zhang, S. Mundlos, L. Christiansen, F. J. Steemers, C. Trapnell, J. Shendure, The single-cell transcriptional landscape of mammalian organogenesis. *Nature* **566**, 496–502 (2019).

41. L. Zane, D. Sibon, C. Legras, J. Lachuer, A. Wierinckx, P. Mehlen, M. H. Delfau-Larue, A. Gessain, O. Gout, C. Pinatel, A. Lancon, F. Mortreux, E. Wattel, Clonal expansion of HTLV-1 positive $CD8^+$ cells relies on *cIAP-2* but not on *c-FLIP* expression. *Virology* **407**, 341–351 (2010).

42. M. Andreatta, A. J. Berenstein, S. J. Carmona, scGate: Marker-based purification of cell types from heterogeneous single-cell RNA-seq datasets. *Bioinformatics* **38**, 2642–2644 (2022).

43. L. C. Garner, A. Amini, M. E. B. FitzPatrick, M. J. Lett, G. F. Hess, M. Filipowicz Sinnreich, N. M. Provine, P. Klenerman, Single-cell analysis of human MAIT cell transcriptional, functional and clonal diversity. *Nat. Immunol.* **24**, 1565–1578 (2023).

44. G. J. J. Kierkels, W. Scheper, A. D. Meringa, I. Johanna, D. X. Beringer, A. Janssen, M. Schiffler, T. Aarts-Riemens, L. Kramer, T. Straetemans, S. Heijhuurs, J. H. W. Leusen, E. San Jose, K. Fuchs, M. Griffioen, J. H. Falkenburg, L. Bongiovanni, A. de Bruin, D. Vargas-Diaz, M. Altelaar, A. J. R. Heck, L. D. Shultz, F. Ishikawa, M. I. Nishimura, Z. Sebestyen, J. Kuball,

Identification of a tumor-specific allo-HLA-restricted gammadeltaTCR. *Blood Adv.* **3**, 2870–2882 (2019).

45. P. M. Benveniste, S. Roy, M. Nakatsugawa, E. L. Y. Chen, L. Nguyen, D. G. Millar, P. S. Ohashi, N. Hirano, E. J. Adams, J. C. Zúñiga -Pflucker, Generation and molecular recognition of melanoma-associated antigen-specific human gammadelta T cells. *Sci. Immunol.* **3**, eaav4036 (2018).

46. L. Luo, W. Liang, J. Pang, G. Xu, Y. Chen, X. Guo, X. Wang, Y. Zhao, Y. Lai, Y. Liu, B. Li, B. Su, S. Zhang, M. Baniyash, L. Shen, L. Chen, Y. Ling, Y. Wang, Q. Liang, H. Lu, Z. Zhang, F. Wang, Dynamics of TCR repertoire and T cell function in COVID-19 convalescent individuals. *Cell Discov.* **7**, 89 (2021).

47. N. Dong, A. Moreno-Manuel, S. Calabuig-Farinas, S. Gallach, F. Zhang, A. Blasco, F. Aparisi, M. Meri-Abad, R. Guijarro, R. Sirera, C. Camps, E. Jantus-Lewintre, Characterization of circulating T cell receptor repertoire provides information about clinical outcome after PD-1 blockade in advanced non-small cell lung cancer patients. *Cancers* **13**, 2950 (2021).

48. X. Bai, Q. Zhang, S. Wu, X. Zhang, M. Wang, F. He, T. Wei, J. Yang, Y. Lou, Z. Cai, T. Liang, Characteristics of tumor infiltrating lymphocyte and circulating lymphocyte repertoires in pancreatic cancer by the sequencing of T cell receptors. *Sci. Rep.* **5**, 13664 (2015).

49. R. K. Sharma, S. V. Boddul, N. Yoosuf, S. Turcinov, A. Dubnovitsky, G. Kozhukh, F. Wermeling, W. W. Kwok, L. Klareskog, V. Malmstrom, Biased TCR gene usage in citrullinated Tenascin C specific T-cells in rheumatoid arthritis. *Sci. Rep.* **11**, 24512 (2021).

50. A. Iyer, D. Hennessey, R. Gniadecki, Clonotype pattern in T-cell lymphomas map the cell of origin to immature lymphoid precursors. *Blood Adv.* **6**, 2334–2345 (2022).

51. M. Shugay, D. V. Bagaev, I. V. Zvyagin, R. M. Vroomans, J. C. Crawford, G. Dolton, E. A. Komech, A. L. Sycheva, A. E. Koneva, E. S. Egorov, A. V. Eliseev, E. Van Dyk, P. Dash, M. Attaf, C. Rius, K. Ladell, J. E. McLaren, K. K. Matthews, E. B. Clemens, D. C. Douek, F. Luciani, D. van Baarle, K. Kedzierska, C. Kesmir, P. G. Thomas, D. A. Price, A. K. Sewell, D.

M. Chudakov, VDJdb: A curated database of T-cell receptor sequences with known antigen specificity. *Nucleic Acids Res.* **46**, D419–D427 (2018).

52. N. Borcherding, A. Vishwakarma, A. P. Voigt, A. Bellizzi, J. Kaplan, K. Nepple, A. K. Salem, R. W. Jenkins, Y. Zakharia, W. Zhang, Mapping the immune environment in clear cell renal carcinoma by single-cell genomics. *Commun. Biol.* **4**, 122 (2021).

53. C. R. Good, M. A. Aznar, S. Kuramitsu, P. Samareh, S. Agarwal, G. Donahue, K. Ishiyama, N. Wellhausen, A. K. Rennels, Y. Ma, L. Tian, S. Guedan, K. A. Alexander, Z. Zhang, P. C. Rommel, N. Singh, K. M. Glastad, M. W. Richardson, K. Watanabe, J. L. Tanyi, M. H. O'Hara, M. Ruella, S. F. Lacey, E. K. Moon, S. J. Schuster, S. M. Albelda, L. L. Lanier, R. M. Young, S. L. Berger, C. H. June, An NK-like CAR T cell transition in CAR T cell dysfunction. *Cell* **184**, 6081–6100.e26 (2021).

54. Z.-Y. Huang, M.-M. Shao, J.-C. Zhang, F.-S. Yi, J. Du, Q. Zhou, F.-Y. Wu, S. Li, W. Li, X.-Z. Huang, K. Zhai, H.-Z. Shi, Single-cell analysis of diverse immune phenotypes in malignant pleural effusion. *Nat. Commun.* **12**, 6690 (2021).

55. H. Xu, J. Jia, Single-cell RNA sequencing of peripheral blood reveals immune cell signatures in Alzheimer's disease. *Front. Immunol.* **12**, 645666 (2021).

56. Z. Wu, S. Gao, N. Watanabe, S. Batchu, S. Kajigaya, C. Diamond, L. Alemu, D. Q. Raffo, X. Feng, P. Hoffmann, D. Stone, A. Ombrello, N. S. Young, Single-cell profiling of T lymphocytes in deficiency of adenosine deaminase 2. *J. Leukoc Biol.* **111**, 301–312 (2022).

57. L. Hoste, L. Roels, L. Naesens, V. Bosteels, S. Vanhee, S. Dupont, C. Bosteels, R. Browaeys, N. Vandamme, K. Verstaen, J. Roels, K. F. A. Van Damme, B. Maes, E. De Leeuw, J. Declercq, H. Aegerter, L. Seys, U. Smole, S. De Prijck, M. Vanheerswynghels, K. Claes, V. Debacker, G. Van Isterdael, L. Backers, K. B. M. Claes, P. Bastard, E. Jouanguy, S. Y. Zhang, G. Mets, J. Dehoorne, K. Vandekerckhove, P. Schelstraete, J. Willems, M.-C. Clinicians, P. Stordeur, S. Janssens, R. Beyaert, Y. Saeys, J. L. Casanova, B. N. Lambrecht, F. Haerynck, S. J. Tavernier, $TIM3^+$ *TRBV11-2* T cells and IFNγ signature in patrolling monocytes and $CD16^+$ NK cells delineate MIS-C. *J. Exp. Med.* **219**, e20211381 (2022).

58. X. Wang, Y. Chen, Z. Li, B. Huang, L. Xu, J. Lai, Y. Lu, X. Zha, B. Liu, Y. Lan, Y. Li, Single-cell RNA-seq of T cells in B-ALL patients reveals an exhausted subset with remarkable heterogeneity. *Adv. Sci.* **8**, 2101447 (2021).

59. B. Liu, X. Hu, K. Feng, R. Gao, Z. Xue, S. Zhang, Y. Zhang, E. Corse, Y. Hu, W. Han, Z. Zhang, Temporal single-cell tracing reveals clonal revival and expansion of precursor exhausted T cells during anti-PD-1 therapy in lung cancer. *Nat. Cancer* **3**, 108–121 (2022).

60. S. Dong, K. J. Hiam-Galvez, C. T. Mowery, K. C. Herold, S. E. Gitelman, J. H. Esensten, W. Liu, A. P. Lares, A. S. Leinbach, M. Lee, V. Nguyen, S. J. Tamaki, W. Tamaki, C. M. Tamaki, M. Mehdizadeh, A. L. Putnam, M. H. Spitzer, C. J. Ye, Q. Tang, J. A. Bluestone, The effect of low-dose IL-2 and Treg adoptive cell therapy in patients with type 1 diabetes. *JCI Insight* **6**, e147474 (2021).

61. R. Furtado, M. Paul, J. Zhang, J. Sung, P. Karell, R. S. Kim, S. Caillat-Zucman, L. Liang, P. Felgner, A. Bauleni, S. Gama, A. Buchwald, T. Taylor, K. Seydel, M. Laufer, F. Delahaye, J. P. Daily, G. Lauvau, Cytolytic circumsporozoite-specific memory $CD4^{+}$ T cell clones are expanded during *Plasmodium falciparum* infection. *Nat. Commun.* **14**, 7726 (2023).

62. J. Li, F. Huang, Q. Ma, W. Guo, K. Feng, T. Huang, Y.-D. Cai, Identification of genes related to immune enhancement caused by heterologous ChAdOx1-BNT162b2 vaccines in lymphocytes at single-cell resolution with machine learning methods. *Front. Immunol.* **14**, 1131051 (2023).

63. R. Di Marco Barros, N. A. Roberts, R. J. Dart, P. Vantourout, A. Jandke, O. Nussbaumer, L. Deban, S. Cipolat, R. Hart, M. L. Iannitto, A. Laing, B. Spencer-Dene, P. East, D. Gibbons, P. M. Irving, P. Pereira, U. Steinhoff, A. Hayday, Epithelia use butyrophilin-like molecules to shape organ-specific γδ T cell compartments. *Cell* **167**, 203–218.e17 (2016).

64. M. Hintz, A. Reichenberg, B. Altincicek, U. Bahr, R. M. Gschwind, A. K. Kollas, E. Beck, J. Wiesner, M. Eberl, H. Jomaa, Identification of (*E*)-4-hydroxy-3-methyl-but-2-enyl pyrophosphate as a major activator for human γδ T cells in *Escherichia coli*. *FEBS Lett.* **509**, 317–322 (2001).

65. P. A. Szabo, H. M. Levitin, M. Miron, M. E. Snyder, T. Senda, J. Yuan, Y. L. Cheng, E. C. Bush, P. Dogra, P. Thapa, D. L. Farber, P. A. Sims, Single-cell transcriptomics of human T cells reveals tissue and activation signatures in health and disease. *Nat. Commun.* **10**, 4706 (2019).

66. M. Andreatta, J. Corria-Osorio, S. Muller, R. Cubas, G. Coukos, S. J. Carmona, Interpretation of T cell states from single-cell transcriptomics data using reference atlases. *Nat. Commun.* **12**, 2965 (2021).

67. B. Garcillan, A. V. Marin, A. Jimenez-Reinoso, A. C. Briones, M. Munoz-Ruiz, M. J. Garcia-Leon, J. Gil, L. M. Allende, E. Martinez-Naves, M. L. Toribio, J. R. Regueiro, γδ T lymphocytes in the diagnosis of human T cell receptor immunodeficiencies. *Front. Immunol.* **6**, 20 (2015).

68. K. Street, D. Risso, R. B. Fletcher, D. Das, J. Ngai, N. Yosef, E. Purdom, S. Dudoit, Slingshot: Cell lineage and pseudotime inference for single-cell transcriptomics. *BMC Genomics* **19**, 477 (2018).

69. S. Gupta, E. Heinrichs, B. G. Novitch, S. J. Butler, Investigating the basis of lineage decisions and developmental trajectories in the dorsal spinal cord through pseudotime analyses. *Development* **151**, dev202209 (2024).

70. K. Wing, S. Sakaguchi, Regulatory T cells exert checks and balances on self tolerance and autoimmunity. *Nat. Immunol.* **11**, 7–13 (2010).

71. B. R. McRitchie, B. Akkaya, Exhaust the exhausters: Targeting regulatory T cells in the tumor microenvironment. *Front. Immunol.* **13**, 940052 (2022).

72. T. Kamada, Y. Togashi, C. Tay, D. Ha, A. Sasaki, Y. Nakamura, E. Sato, S. Fukuoka, Y. Tada, A. Tanaka, H. Morikawa, A. Kawazoe, T. Kinoshita, K. Shitara, S. Sakaguchi, H. Nishikawa, PD-1^{+} regulatory T cells amplified by PD-1 blockade promote hyperprogression of cancer. *Proc. Natl. Acad. Sci. U. S. A.* **116**, 9999–10008 (2019).

73. T. Deng, H. Wang, C. Yang, M. Zuo, Z. Ji, M. Bai, T. Ning, R. Liu, J. Wang, S. Ge, L. Zhang, Y. Ba, H. Zhang, Single cell sequencing revealed the mechanism of PD-1 resistance affected by

the expression profile of peripheral blood immune cells in ESCC. *Front. Immunol.* **13**, 1004345 (2022).

74. G. Qiu, J. Liu, Q. Cheng, Q. Wang, Z. Jing, Y. Pei, M. Zhao, J. Wang, J. Y. Guo, J. Zhang, Impaired autophagy and defective T cell homeostasis in mice with T cell-specific deletion of receptor for activated C kinase 1. *Front. Immunol.* **8**, 575 (2017).

75. O. Ballek, J. Valecka, M. Dobesova, A. Brouckova, J. Manning, P. Rehulka, J. Stulik, D. Filipp, TCR triggering induces the formation of Lck-RACK1-actinin-1 multiprotein network affecting Lck redistribution. *Front. Immunol.* **7**, 449 (2016).

76. X. Zhang, J. Zhang, W. Zhao, X. Dong, P. Xin, X. Liu, X. Li, Z. Jing, Z. Zhang, C. Kong, X. Yu, Long non-coding RNA LINC02446 suppresses the proliferation and metastasis of bladder cancer cells by binding with EIF3G and regulating the mTOR signalling pathway. *Cancer Gene Ther.* **28**, 1376–1389 (2021).

77. M. S. Davey, C. R. Willcox, A. T. Baker, S. Hunter, B. E. Willcox, Recasting human Vδ1 lymphocytes in an adaptive role. *Trends Immunol.* **39**, 446–459 (2018).

78. S. Mensurado, R. Blanco-Dominguez, B. Silva-Santos, The emerging roles of γδ T cells in cancer immunotherapy. *Nat. Rev. Clin. Oncol.* **20**, 178–191 (2023).

79. L. Song, D. Cohen, Z. Ouyang, Y. Cao, X. Hu, X. S. Liu, TRUST4: Immune repertoire reconstruction from bulk and single-cell RNA-seq data. *Nat. Methods* **18**, 627–630 (2021).
